# The activated CD36-Src axis promotes lung adenocarcinoma cell proliferation and actin remodeling-involved metastasis in high-fat environment

**DOI:** 10.1038/s41419-023-06078-3

**Published:** 2023-08-23

**Authors:** Li-Zhong Liu, Bowen Wang, Rui Zhang, Zangshu Wu, Yuxi Huang, Xiaoyang Zhang, Jiaying Zhou, Junbo Yi, Jian Shen, Ming-Yue Li, Ming Dong

**Affiliations:** 1 Department of Physiology, School of Basic Medical Sciences, Shenzhen University Medical School, Shenzhen University, Shenzhen, 518055 Guangdong China; 2 GuangZhou National Laboratory, Guangzhou International Bio Island, No. 9 XingDaoHuanBei Road, Guangzhou, 510005 Guangdong China; 3 Faculty of Medicine, The Chinese University of Hong Kong, Hong Kong, China; 4 Present Address: Guangdong Medical Academic Exchange Center, Yuexiu District, Guangzhou, Guangdong China

**Keywords:** Non-small-cell lung cancer, Non-small-cell lung cancer

## Abstract

Obesity/overweight and lipid metabolism disorders have become increased risk factors for lung cancer. Fatty acid translocase CD36 promotes cellular uptake of fatty acids. Whether and how CD36 facilitates lung adenocarcinoma (LUAD) growth in high-fat environment is unknown. Here, we demonstrated that palmitic acid (PA) or high-fat diet (HFD) promoted LUAD cell proliferation and metastasis in a CD36-dependent manner. Mechanistically, CD36 translocated from cytoplasm to cell membrane and interacted with Src kinase upon PA stimulation in human LUAD cells. Akt and ERK, downstream of Src, were then activated to mediate LUAD cell proliferation and metastasis. Furthermore, PA treatment promoted CD36 sarcolemmal translocation, where it activated Rac1 and upregulated MMP-9 through Src-Akt/ERK pathway, resulting in redistribution of cortactin, N-WASP and Arp2/3, and finally led to occurrence of finger-like protrusions of actin on cell surface to enhance cell metastasis. Compared with normal-chew diet (NCD) mice, the HFD group exhibited higher level of blood free fatty acid (FFA) and cholesterol (TC), developed larger xenograft LUAD tumors and enhanced tumor cell metastatic potential, which were accompanied by obvious sarcolemmal actin remodeling and were blocked by simultaneous CD36 knockdown in LUAD cells. Consistently, xenografted and tail vein-injected scramble-RNA-A549 cells but not CD36-shRNA-A549 in HFD mice formed metastatic LUAD tumors on the lung. CD36 inhibitor SSO significantly inhibited LUAD cell metastasis to the lung. Collectively, CD36 initiates Src signaling to promote LUAD cell proliferation and actin remodeling-involved metastasis under high-fat environment. Our study provides the new insights that CD36 is a valid target for LUAD therapy.

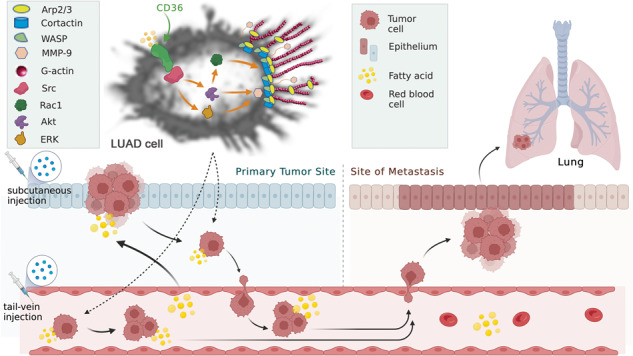

## Introduction

With high morbidity and mortality, lung cancer is still the most common cancer worldwide [1]. Specially, the incidence rate of lung adenocarcinoma (LUAD), one of the non-small cell lung cancers (NSCLC), is still rising [2, 3]. The main reason for the high incidence of lung cancer is the result of the joint action of environmental and genetic factors [4], and in addition to smoking, other factors can still not be ignored [2]. Obesity/overweight and lipid metabolism disorders are becoming more and more popular and caused many health problems, including the increased risk and poor prognosis of many cancers [5–7]. One of the main characteristics of obesity is the increase of free fatty acids (FFAs) in blood, and FFAs are essential for tumor growth and development [8–11]. Although smoking can lead to general weight loss, studies find that central obesity which particularly concurrent with low body mass index, large waist circumference, hyperlipidemia and muscle loss, may help identify high-risk populations for lung cancer [12–14].

Fatty acid translocase CD36 is involved in the cellular uptake of long-chain fatty acids and oxidized low-density lipoprotein [15]. Fatty acid (FA) uptake and lipid metabolism are essential cellular processes to promote tumorigenesis and tumor progression [16], and CD36 is involved in this process [17, 18]. Several studies also suggest that CD36 is over-expressed and drives progression in several cancers [19–23]. Our previous studies have demonstrated that Src family member Fyn formed CD36-Fyn complex to regulate fatty acid uptake in skeletal muscle cells [24]. Increased expression of Src and its activity, bur not Fyn, have been reported in NSCLC, particularly in LUAD [25]. Activated Src is involved in inducing lung cancer cell migration and invasion [26]. In this context, we speculate that CD36 might promote LUAD development via Src.

LUAD cell migration and invasion serve as critical parameters of the metastasis cascade leading to high mortality rate [27, 28]. Dynamic changes in actin filaments are involved in a wide variety of cellular processes including cell motility [29]. The rearranged actin filaments, also termed as actin-remodeling [30], is mainly regulated by members of the Rho family (Rho GTPases, including Rho, Cdc42, Rac, and its isoforms) signals to form discrete structures at the cell periphery and mediate attachment to the substratum [31]. Those discrete structures are essential for cell migration and invasion [31]. Actin-remodeling occurs when dorsal circular ruffles (DCRs) formed at the leading edge of cells, provides a relaxation of static actin structures to form pliable membrane protrusions [30, 32]. Rho GTPases, like Rac1 or Cdc42, are required for DCRs formation [33]. DCRs are also able to secrete matrix metalloproteinases (MMPs) and enriched with actin assembly proteins, such as Arp2/3, WASP, and cortactin, revealing their potential role for the cell metastasis onset [32]. However, when LUAD cells facing with a high-fat environment, the role and regulation of CD36 on LUAD cell proliferation and actin remodeling-involved metastasis is poorly understood. We here explore the relationship between lipid metabolism disorders and the LUAD occurrence and development, clarify whether high fat affects the proliferation and metastasis of LUAD cells, and further reveal the molecular mechanism of CD36-regulated LUAD development beyond.

## Results

### CD36 was indispensable for PA-induced LUAD cell proliferation and metastasis

To examine the effect of PA on LUAD cell proliferation, migration and invasion, both NCI-H23 and A549 cells were treated with different concentration of PA (molecular ratio of PA:BSA = 1:3) for different time to optimize the condition of PA treatment. For NCI-H23 cell, 10–500 μM PA with 24 h incubation or 0.1–100 μM PA with 48 h incubation significantly enhanced cell viability, while 500 μM PA produced lipotoxicity after 48 h. For A549 cells, 1–50 μM PA increased cell viability after 24 h or 48 h, but 500 μM PA showed lipotoxicity even at 24 h (Fig. S1A and S1B). In the BrdU experiment, NCI-H23 or A549 cells proliferation was significantly enhanced after 6 h of stimulation with different concentrations of PA (Fig. S1C). The above results showed that cells incubated with 500 μM PA for a short time (≤6 h) did not exhibit toxicity. Combined our results with the previous reports [19], in the following experiments, 500 μM PA was used in a shorter duration time (≤1 h) and 10 μM PA was used in a longer duration time (≥6 h) to obtain the best observed effect for both NCI-H23 and A549 cells. With proper concentration and incubation time of PA, we found both cell migration (Fig. S1D and S1E) and cell invasion (Fig. S1F) were enhanced obviously.

**Fig. 1:**
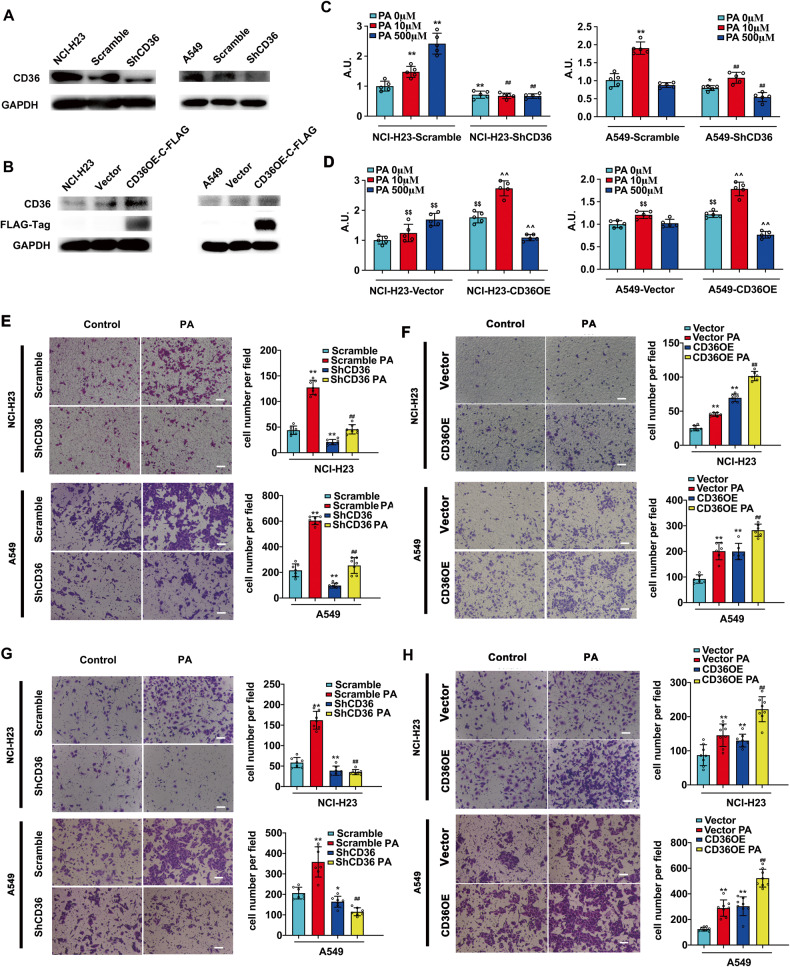
CD36 was required for PA-induced LUAD cell proliferation and metastasis. **A** CD36 knockdown and **B** CD36 overexpression LUAD cell lines construction. Lentivirus transfection was performed to construct cell lines of NCI-H23-Scramble, NCI-H23-ShCD36, A549-Scramble, A549-ShCD36, NCI-H23-Vector, NCI-H23-CD36OE-FLAG, A549-Vector, and A549-CD36OE-FLAG. The efficiency of knockdown or overexpression was confirmed by CD36 protein examination. **C**, **D** Cell proliferation assay. Cells were treated with PA for 6 h, then BrdU assay was performed. 0 μM PA (non-treatment) 6 h was set up as 1, *p < 0.05 and **p < 0.01 vs. scramble, ^#^*p* < 0.05 and ^##^*p* < 0.01 vs^.^ scramble+PA, *n* = 6. ^$^*p* < 0.05 and ^$$^*p* < 0.01 vs. vector, ^^^*p* < *0*.05 and ^^^^*p* < 0.01 vs. vector+PA^,^ *n* = 6. **E**, **F** Cell migration and **G**, **H** invasion assay^.^ Cells were treated with 10 μM PA for 24 h were applied for trans-well assay to detect cell migration and invasion respectively. Images were taken using light microscopy (scale bar: 50 μm). The numbers of migration or invasion cells in seven randomly selected fields were counted and the average number of cells in one field was calculated and expressed as the mean ± SD. *p < 0.05 and **p < 0.01 vs scramble/vector respectively; ^#^*p* < 0.05 and ^##^*p* < 0.01 vs scramble/vector+PA respectively, *n* = 3.

To determine whether the effect of PA depended on CD36, we constructed CD36 knockdown LUAD cell lines (Fig. 1A) and CD36 overexpression LUAD cell lines respectively (Fig. 1B). Both basal and PA-induced lung cell proliferation could be significantly inhibited by CD36 knockdown (Fig. 1C). CD36 overexpression promoted cell proliferation even there was no PA stimulation (Fig. 1D). Surprisingly, contrary to the effect of 10μM PA, 500μM PA elicited lipotoxicity in CD36 overexpressed cells (Fig. 1D), which might due to CD36 overexpression caused excessive intake of fatty acids (lipotoxicity) after high concentration PA treatment. Consistently, CD36 knockdown inhibited 10 μM PA-induced cell migration (Fig. 1E, Fig. S2A) and cell invasion (Fig. 1G). CD36 overexpression not only exhibited a PA-like effect in basal state but also facilitated PA effect in promoting cell migration (Fig. 1F, Fig. S2B) and invasion (Fig. 1H). These changes further validated the indispensable role of CD36 in PA-induced LUAD carcinogenesis.

### CD36 translocation to LUAD cell membrane was enhanced by PA stimulation

**Fig. 2:**
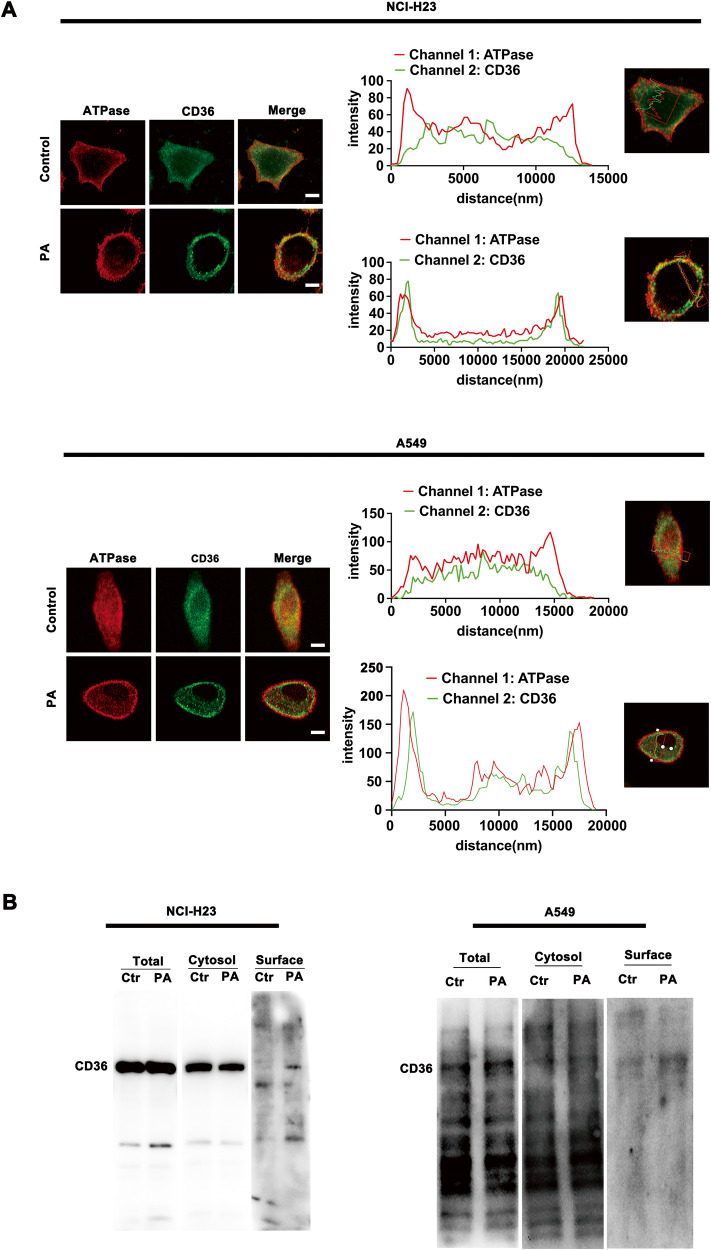
CD36 translocated to plasma membrane of LUAD cell upon PA stimulation. **A** CD36 distribution on the cell membrane was enhanced by PA treatment. NCI-H23 and A549 cells were treated with or without 500 μM PA for 0.5 h respectively. Cells were double stained for CD36 (green) and Na-K ATPase (red) respectively. Bar, 5 μm. The images were representative of three experiments. Linear scan the fluorescence intensity was done to determine the CD36 distribution by scanning the cell cross section along the straight line with Zeiss microscope software. **B** PA increased the content of CD36 on plasma membrane. NCI-H23 and A549 cells were treated with or without 500 μM PA for 30 min. Subcellular fractionation was performed to purify the plasma and cytosol fractions. Equal amount proteins were subjected to immunoblot for CD36 with corresponding antibodies. The images were representative of three experiments (Ctr: non-PA treatment).

CD36 needs to insert into the cell membrane before it combines with and transport extracellular FA. Na-K ATPase was used to outline the cell membrane. Immunofluorescence (IF) results showed that CD36 largely dispersed in cytosol and less located on the cell membrane in basal state. Under PA stimulation, the sarcolemmal CD36 increased obviously (Fig. 2A). Furthermore, by linear scan the fluorescence intensity of cross cell section with Zeiss microscope software, it confirmed that CD36 translocation to the cell membrane was enhanced by PA treatment, which evidenced by the fluorescence signal peak of CD36 and Na-K ATPase occurred almost at the same site (Fig. 2A). We further extracted cell membrane proteins and found that sarcolemmal CD36 increased to a certain extent in PA-treated LUAD cells (Fig. 2B).

### Sarcolemmal CD36 interacted with Src kinase to initiate PA-induced Src/Akt/ERK1/2 kinase activation in LUAD cells

**Fig. 3:**
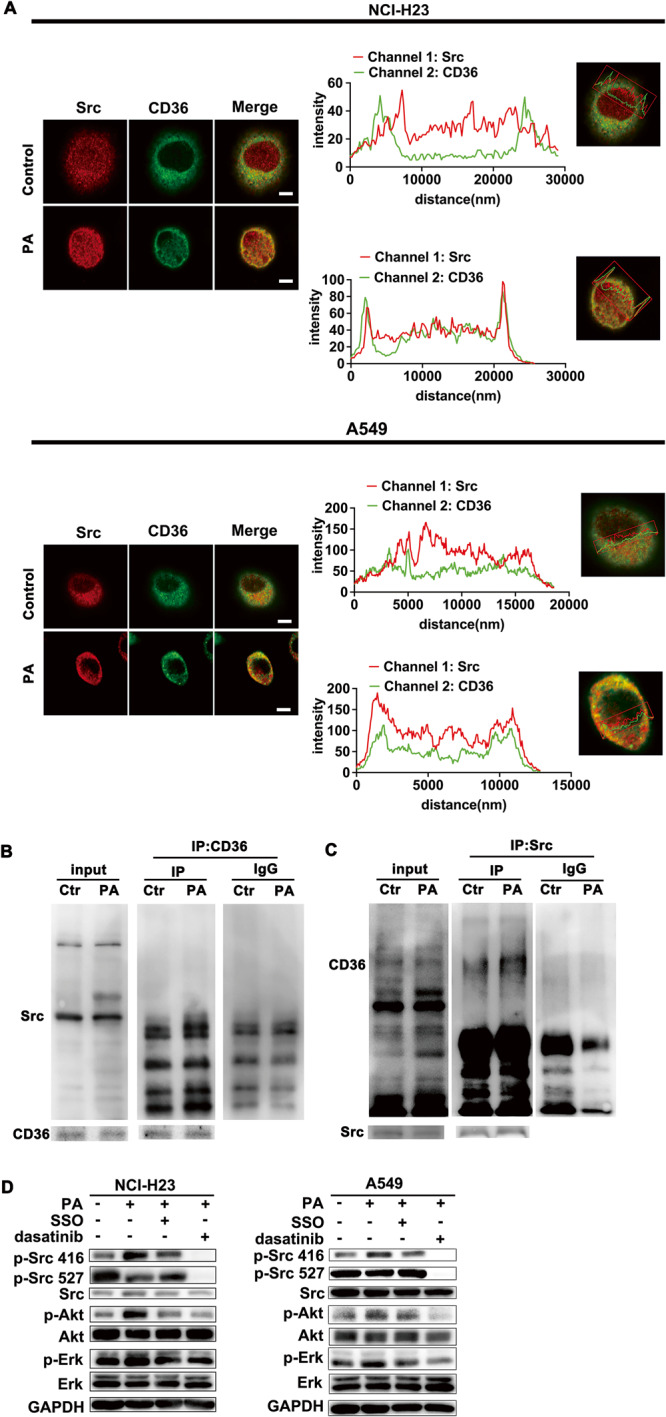
Sarcolemmal CD36 interacted with Src kinase to initiate PA-induced Src/Akt/ERK1/2 kinase activation in LUAD cells. **A** PA promoted co-localization between sarcolemmal CD36 and Src. NCI-H23 and A549 cells were treated with or without 500 μM PA for 0.5 h respectively. Cells were double stained for Src (red) and CD36 (green) respectively. Bar, 5 μm. The images were representative of three experiments. Linear scan the fluorescence intensity was done to determine the distribution of Src and CD36 by scanning the cell cross section along the straight line with Zeiss microscope software. **B**, **C** PA promoted the interaction between CD36 and Src. NCI-H23 cells were treated with or without 500 μM PA for 0.5 h. Total cell lysis (input) was detected with indicated antibodies. CD36 was immunoprecipitated by its own antibody and then the immunoprecipitates were examined with Src antibodies (Ctr: non-PA treatment). Similarly, Src immunoprecipitates (IP by Src antibody) were examined with CD36 antibody. **D** CD36 initiated PA-induced Src/Akt/ERK1/2 signaling pathway. NCI-H23 and A549 cells were treated with or without 500 μM PA for 0.5 h, or cells were pretreated with 50 μM SSO (a fatty acid analog as CD36 blocker) and 10 μM Dasatinib (Src inhibitor) for 15 min respectively, followed by 500 μM PA treatment for another 30 min. Then proteins were examined with indicated antibodies.

The above experiments indicated that CD36 may not only mediate FA uptake, but also initiate carcinogenic signaling events in PA-treated LUAD cells proliferation and metastasis. We then try to further verify CD36 and Src kinase relationship. IF results showed PA treatment promoted CD36 and Src colocalization (Fig. 3A). Combined with the fact that PA induced CD36 translocation to the cell membrane (Fig. 2), we concluded that PA stimulation enhanced colocalization of Src and sarcolemmal CD36 (Fig. 3A). Furthermore, by linear scan the fluorescence intensity of cross cell section, the spatial colocalization of Src with CD36 was found to be increased under PA treatment, which evidenced by the fluorescence signal peak of CD36 and Src occurred almost at the same site (Fig. 3A). Immunoprecipitation results confirmed that CD36 interacted with Src under PA treatment (Fig. 3B, C). The phosphorylation of Src-Tyr416 increased while Src-Tyr527 decreased, indicating that Src could be activated upon PA treatment (Fig. 3D). At the same time, the activity of Akt and ERK1/2 increased too (Fig. 3D). SSO, a fatty acid analog as CD36 blocker, effectively blocked the kinases activation induced by PA (Fig. 3D). Moreover, Src kinase inhibitor dasatinib effectively blocked PA-induced activation of Src, Akt and ERK1/2 (Fig. 3D), suggesting that Src kinase located upstream of Akt and ERK1/2 to regulate their activity. Therefore, PA-mediated CD36 sarcolemmal migration enhanced CD36 colocalization with Src kinase, leading to Src kinase activation, which followed by Akt and ERK1/2 activation. The results demonstrated that CD36 was indispensable for PA to activate Src/Akt/ERK1/2 signal axis.

### CD36 sarcolemmal distribution and the followed Src activation were indispensable for PA-induced actin remodeling

**Fig. 4:**
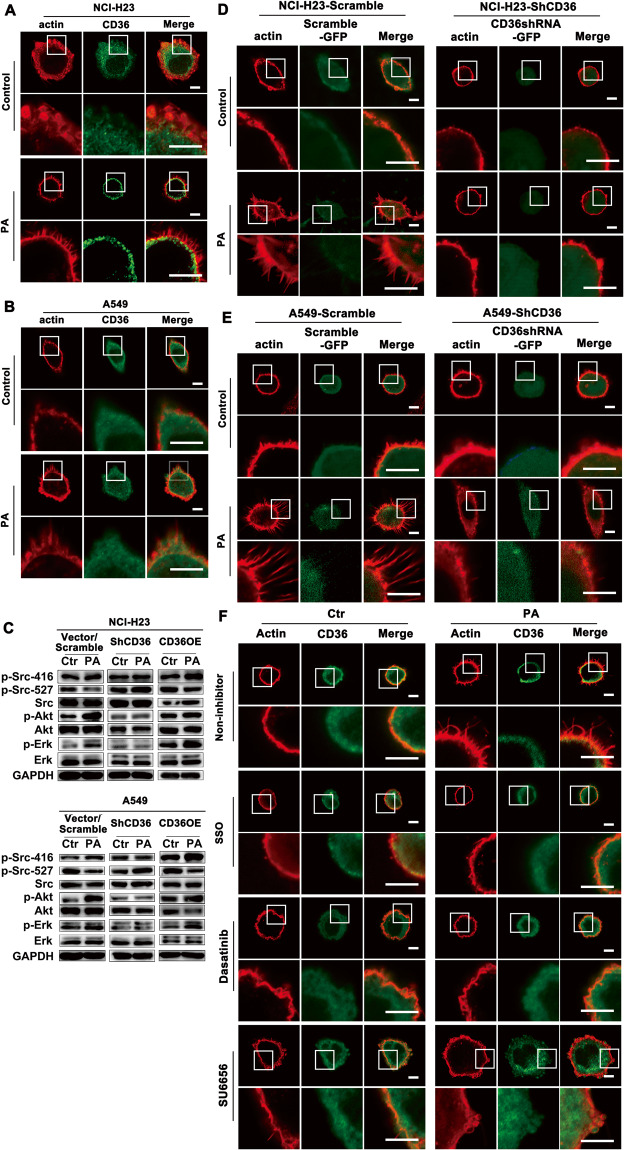
CD36-Src signal was required for PA-induced actin remodeling. **A**, **B** CD36 distribution and actin remodeling upon PA stimulation. NCI-H23 and A549 cells were treated with or without 500 μM PA for 0.5 h, respectively. Then cells were double stained for actin (red) and CD36 (green) respectively. Bar, 5 μm. The images were representative of three experiments. **C** PA upregulated the activation of Src/Akt/ERK1/2 via CD36. Cells were treated with or without 500 μM PA for 0.5 h. Untreated cells were set up as controls. Then proteins were examined with corresponding antibodies as indicated. Ctr: non-PA treatment. **D**, **E** CD36 was required for PA-induced actin remodeling. Scramble and ShCD36 LUAD cells were treated with or without 500 μM PA for 0.5 h. Cells were stained for actin (red). Cells given green fluorescence contained the GFP protein, proving the stable transfected cells as indicated in the *Materials and Methods*. Bar, 5 μm. The images were representative of three experiments. **F** CD36-Src signal was required for PA-induced actin remodeling. NCI-H23 and A549 cells were treated with or without 500 μM PA for 0.5 h, or cells were pretreated with 50 μM SSO (a fatty acid analog as CD36 blocker), 100 nM SU6656 (Src inhibitor) and 10 μM Dasatinib (Src inhibitor) respectively for 15 min, followed by 500 μM PA treatment for another 0.5 h. Then cells were double stained for actin (red) and CD36 (green) respectively. Bar, 5 μm. The images were representative of three experiments.

The dynamic change of actin structure is required for cell metastasis. Actin-remodeling are involved in a wide variety of cellular processes including cell motility [29]. In order to find out whether CD36 played an important role in PA-induced actin remodeling, we firstly observed the dynamic change of actin structure in LUAD cells under PA treatment. IF staining showed that, compared with PA-untreated cells, actin just beneath plasma membrane underwent structural remodeling to protrude out of the membrane and formed finger-like protrusion in PA-treated LUAD cells, where the sarcolemmal CD36 increased obviously (Fig. 4A, B). The activity of Src, Akt and ERK1/2 kinase was increased in PA-treated cells. The kinase activity could be further activated in CD36OE cells while blocked in ShCD36 cells (Fig. 4C), indicating that CD36 was pivotal for PA to activate Src/Akt/ERK1/2 signal axis. Consistence with this, actin-remodeling induced by PA disappeared when CD36 was knockdown (Fig. 4D, E). And PA could not induce finger like actin-remodeling on the cell surface when CD36 inhibitor SSO or Src inhibitor dasatinib/SU6656 was applied (Fig. 4F). The above experiments proved that PA-induced CD36 sarcolemmal translocation and Src activation regulated actin structural remodeling.

### CD36 mediated PA-induced actin remodeling through Rac1

**Fig. 5:**
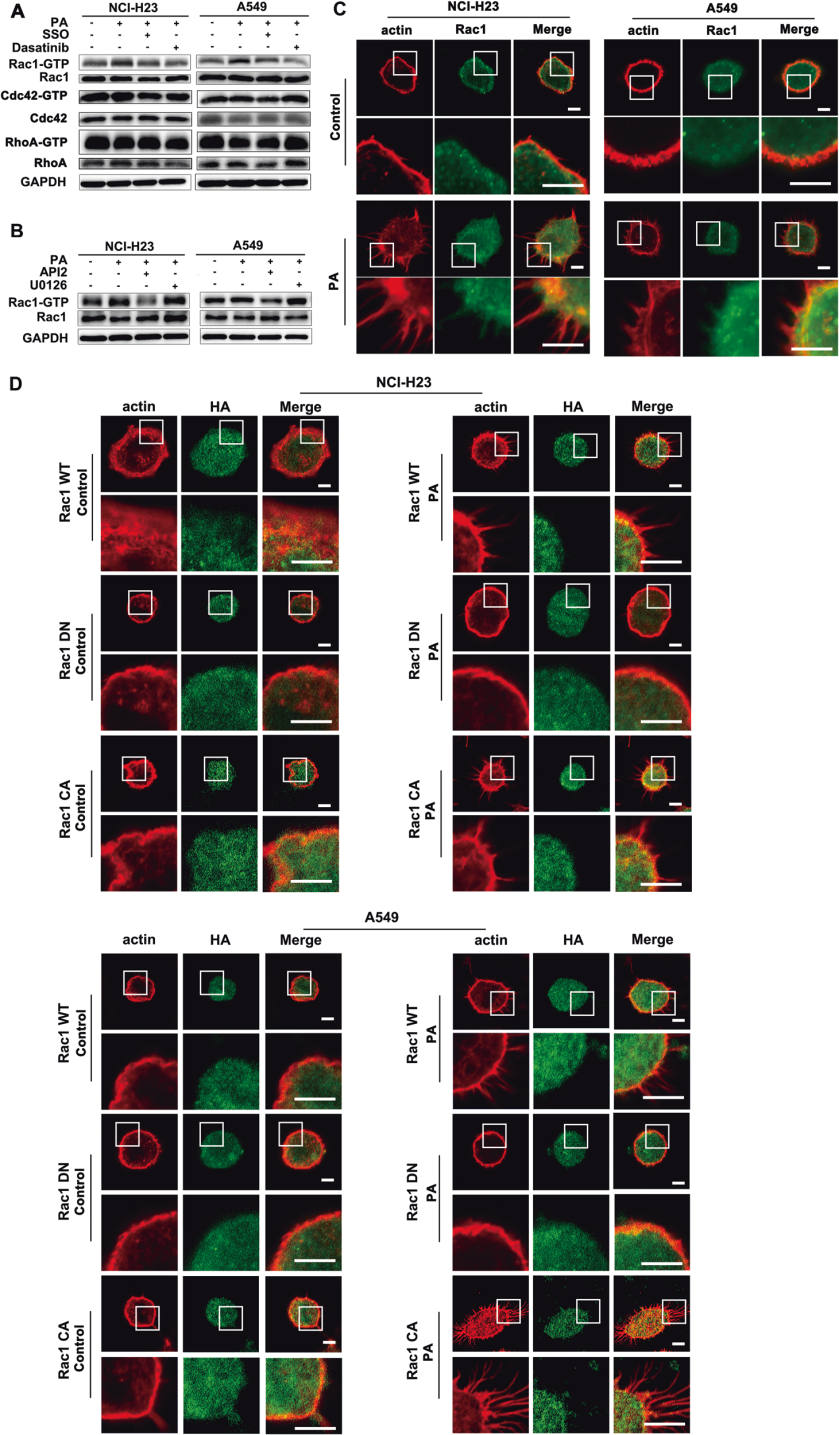
Rac1 participated PA-induced actin-remodeling in LUAD cells. **A** PA activated Rac1 in a CD36-Src signal-dependent manner. NCI-H23 and A549 cells were treated with or without 500 μM PA for 0.5 h, or cells were pretreated with 50 μM SSO (a fatty acid analog as CD36 blocker), and 10μM Dasatinib (Src inhibitor) for 15 min, respectively, which was followed by 500 μM PA treatment for another 0.5 h. The GTP binding Rac1, Cdc42, and RhoA were detected with kits as mentioned in *Materials and Methods* and their expression was examined with antibodies as indicated. **B** Akt kinase but not ERK regulated Rac1 activity. NCI-H23 and A549 cells were treated with or without 500 μM PA for 0.5 h, or cells were pretreated with MEK/ERK inhibitor U0126 (10 μM), and Akt inhibitor API2 (20 μM) for 15 min respectively, which was followed by 500 μM PA treatment for another 0.5 h. GTP binding Rac1 and its expression were detected. **C** Rac1 colocalized with the finger like actin remodeling after PA treatment. NCI-H23 and A549 cells were treated with or without 500 μM PA for 0.5 h respectively. Cells were double stained for actin (red) and Rac1 (green) respectively. Bar, 5 μm. The images were representative of three experiments. **D** Rac1 was required for PA-induced actin remodeling. NCI-H23 and A549 cells were transiently transfected with the HA-tagged wild type Rac1 (Rac1 WT), dominant negative Rac1 (Rac1 DN) and constitutively active Rac1 (Rac1 CA) respectively. After 48 h of transfection, cells were treated with or without 500 μM PA for 0.5 h. Then cells were double stained for actin (red) and HA (green) respectively. Bar, 5 μm. The images were representative of three experiments.

Rho GTPases play an important role in the regulation of actin structure. In order to verify whether Rho family members participate in PA-induced actin remodeling in a CD36-Src signal dependent manner, we detected the change of PA-induced expression/activity of Rho family members Rac1, RhoA and Cdc42 as indicated in Fig. 5A. Although the expression of the three Rho family members did not change obviously, the activity of Rac1 but not RhoA or Cdc42 increased significantly after PA treatment, suggesting that Rac1 was most likely to participate in the PA-induced actin remodeling in LUAD cells (Fig. 5A). CD36 inhibitor SSO, Src inhibitor dasatinib and Akt inhibitor API2 could effectively inhibit PA-induced Rac1 activation, respectively (Fig. 5A, B). While ERK inhibitor U0126 could not block the effect of PA on Rac1 (Fig. 5B). Thus, PA-induced Rac1 activation was in a CD36-Src-Akt dependent manner in LUAD cells (Fig. 5A, B). Next, we examined the distribution of Rac1, RhoA and Cdc42 and their association with actin protrusions upon PA stimulation. The results showed it was Rac1 (Fig. 5C) but not RhoA and Cdc42 (Fig. S3) that partially colocalized with the finger like actin remodeling on the surface of LUAD cells. Next, the LUAD cells were transiently transfected with HA-tagged Rac1 WT, Rac1 DN, and Rac1 CA respectively and then were immune-stained with HA antibody (cells with green fluorescence exhibited successful transfection) and actin antibody. This investigation proved that the PA effect on actin remodeling disappeared in LUAD cells transfected with the Rac1 DN plasmid, while PA treatment promoted actin structural remodeling both in the Rac1 WT or Rac1 CA transfected LUAD cells (Fig. 5D). Therefore, Rac1 activity enhanced by PA through the CD36-Src-Akt signaling pathway played the key role in mediating actin-remodeling and promoting tumor metastasis.

### PA stimulated redistribution of MMP-9, α-tubulin, Arp2/3 and cortactin closely related to actin remodeling

**Fig. 6:**
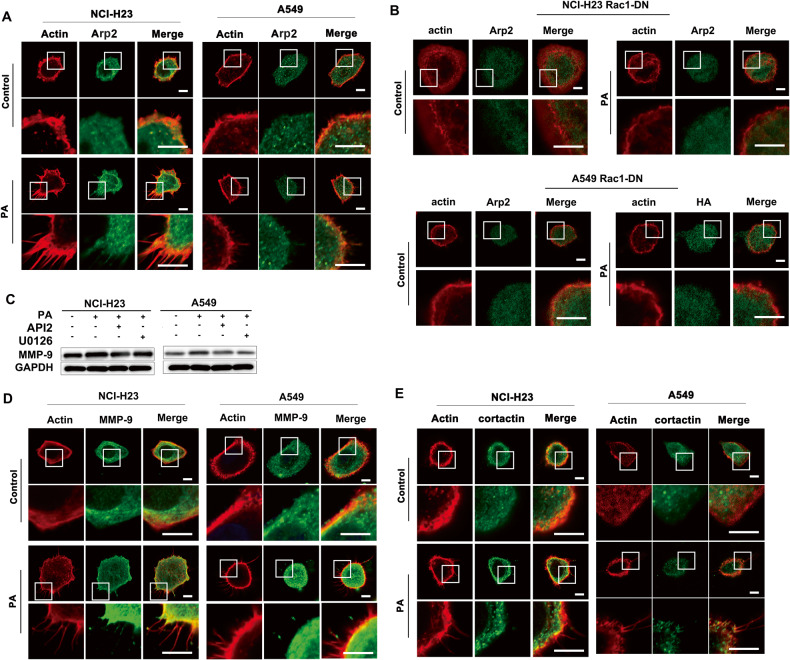
PA stimulated redistribution of Arp2, MMP-9 and cortactin closely related to actin remodeling. **A** PA treatment led to redistribution of Arp2. NCI-H23 and A549 cells were treated with or without 500 μM PA for 0.5 h respectively. Cells were double stained for actin (red) and Arp2 (green), respectively. Bar, 5 μm. The images were representative of three experiments. **B** Rac1 regulated redistribution of Arp2. NCI-H23 and A549 cells stable transfected with Rac1-DN were treated with or without 500 μM PA for 0.5 h respectively. Cells were double stained for actin (red) and Arp2 (green), respectively. Bar, 5 μm. The images were representative of three experiments. **C** PA regulated MMP-9 expression via Akt and ERK. NCI-H23 and A549 cells were treated with or without 10 μM PA for 24 h, or cells were pretreated with MEK/ERK inhibitor U0126 (10 μM), and Akt inhibitor API2 (20 μM) for 30 min, respectively, which was followed by 10 μM PA treatment for another 24 h. MMP-9 and GAPDH expression were determined by western blot. **D**, **E** PA treatment led to redistribution of MMP-9 and cortactin. NCI-H23 and A549 cells were treated with or without 500 μM PA for 0.5 h respectively. Cells were double stained for actin (red), MMP-9 (green) and cortactin (green), respectively. Bar, 5 μm. The images were representative of three experiments.

Next, we examined the proteins related to actin assembly [34]. Our results showed that Arp2 and Arp3 involved in actin remodeling formation were relocated at the root of the formed actin protrusion under PA stimulation (Figs. 6A and S4A). Further investigation proved that Arp2 relocation disappeared in Rac1 DN plasmid-transfected LUAD cells under PA treatment, indicating that Rac1 was upstream of Arp2 in PA-inducing actin remodeling (Fig. 6B). MMP-9 expression was significantly increased under PA stimulation, and both Akt inhibitor API2 and ERK inhibitor U0126 could effectively inhibited MMP-9 induced by PA respectively (Fig. 6C). Thus, MMP-9 induced by PA in LUAD cells was in a CD36-Src-ERK/Akt dependent manner. The further investigation showed that PA induced MMP-9 locating at the tip of the actin protrusions on the cell membrane surface (Fig. 6D). This is consistent with the characteristics of invasive processes since MMP-9 can degrade the extracellular matrix to help cell penetrate into the extracellular matrix [34]. The distribution of cortactin also changed to the cell membrane and gathered at the root of actin protrusions under PA stimulation, and some cortactin colocalized with actin protrusions (Fig. 6E). The same tendency happened on N-WASP distribution (Fig. S4B). In addition, the distribution of tubulin was mainly in the root of the protrusion (Fig. S4C), which provided some support for the protrusion [35]. Given the evidence that CD36 mediated PA-induced actin remodeling (Fig. 4), we could speculate that PA changed the distribution of MMP-9, tubulin, N-WASP, cortactin and Arp2/3 through CD36-Src-Akt/ERK signaling pathway to trigger actin structural remodeling in LUAD cells, and finally promote the invasion of tumor cells.

### CD36 was required for in situ tumor formation and pulmonary metastasis in HFD-fed nude mice

**Fig. 7:**
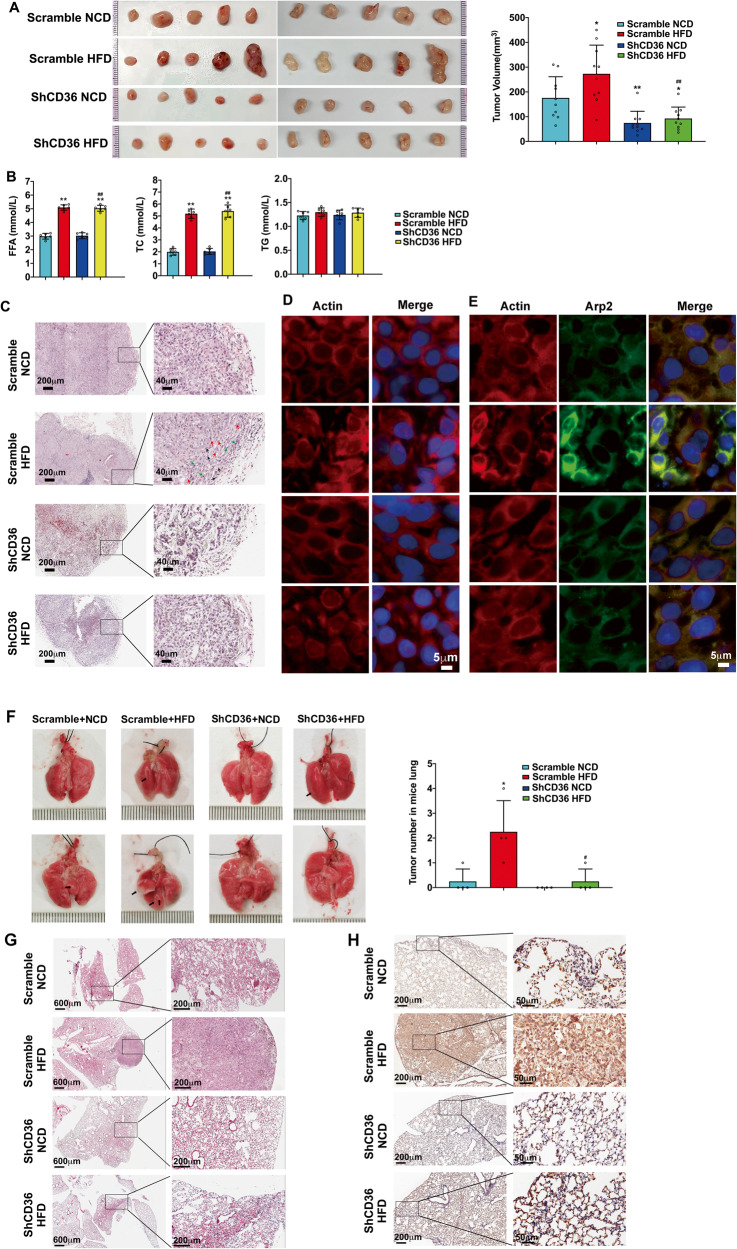
CD36 knockdown in LUAD cells inhibited xenograft tumor growth, aggressiveness and metastasis. **A** CD36 knockdown in LUAD cells inhibited xenograft tumor growth. The nude mice fed with NCD and HFD were subcutaneously transplanted with A549-Scramble and A549-ShCD36 cells respectively. The tumor was collected 5 weeks later and the size of tumor was measured (*n* = 10, *p < 0.05 and **p < 0.01 vs. Scramble NCD; ^##^*p* < 0.01 vs. Scramble HFD). **B** Measurement of plasma free fatty acids (FFA), total cholesterol (TC) and triglyceride (TG). FFA, TC and TG in mice blood were measured with kits according to the instructions. **p < 0.01 vs. Scramble NCD, ^##^*p* < 0.01 vs. ShCD36 HFD, *n* = 5. **C** CD36 knockdown in LUAD cells inhibited xenograft tumor aggressiveness. Subcutaneous tumor sections from A549-Scramble NCD, A549-Scramble HFD, A549-ShCD36 NCD, or A549-ShCD36 HFD were stained with H&E and then examined. The poorly differentiated and more aggressive cells in A549-Scramble HFD tumors (Spindle cells (red arrows), cells with high nuclear-cytoplasm ratio (green arrows), mitotic cells (black arrows) were indicated. **D** CD36 knockdown in LUAD cells inhibited actin-remodeling in subcutaneous tumor cells of HFD-fed mice. Subcutaneous tumor sections were stained for actin (red). The representative IF staining image of tumors generated from the mice of A549-Scramble NCD, A549-Scramble HFD, A549-ShCD36 NCD, or A549-ShCD36 HFD were shown. Bar, 5 μm. The images were representative of three experiments. **E** CD36 knockdown in LUAD cells inhibited colocalization between Arp2 and actin in subcutaneous tumor cells of HFD-fed mice. Subcutaneous tumor sections were double stained for actin (red) and Arp2 (green) respectively. The representative IF staining image of tumors generated from the mice of A549-Scramble NCD, A549-Scramble HFD, A549-ShCD36 NCD, or A549-ShCD36 HFD were shown. Bar, 5 μm. The images were representative of three experiments. **F** Detection of lung metastasis of subcutaneous tumor cells. After subcutaneous tumor removal, the mice were continued to be fed with NCD or HFD for another 12 weeks and then sacrificed. Lungs of nude mice were collected and LUAD cell metastasis to lung were examined and lung metastatic nodules were calculated. Lung surface nodules were indicated as black arrows. Data were mean ± SD, *p < 0.05 vs. Scramble NCD, ^#^*p* < 0.05 vs. Scramble HFD, *n* = 4. **G** Pathological analysis of the lungs from subcutaneous tumor removal mice. Lung tissue sections from mice of A549-Scramble NCD, A549-Scramble HFD, A549-ShCD36 NCD, or A549-ShCD36 HFD were stained with H&E and examined. **H** IHC staining of the lungs from subcutaneous tumor removal mice. Lung tissue sections from mice of A549-Scramble NCD, A549-Scramble HFD, A549-ShCD36 NCD, or A549-ShCD36 HFD were stained with CD36 and examined.

We generated subcutaneous xenograft models to confirm the tumor-promoted function of CD36 in HFD-fed nude mice. In Fig. 7A, tumor volume derived from A549-scramble cell transplanted HFD-fed mice was significantly larger than that of NCD-fed mice (*p* < 0.05). A549-ShCD36 cells transplanted into NCD-fed mice and even into HFD-fed mice exhibited markedly decreased tumor growth (*p* < 0.01 and *p* < 0.05 respectively). Moreover, tumor volume derived from A549-ShCD36 cells transplanted into HFD-fed mice was significantly smaller than that of A549-Scramble cells transplanted into HFD-fed mice (*p* < 0.01). These results indicated that knockdown of CD36 significantly counteracted the facilitation of HFD on LUAD tumor growth. High fat environment in the HFD-fed mice were confirmed by the elevated blood FFA and TC (Fig. 7B). Pathologic analysis revealed that relative to A549-Scramble tumors in NCD-fed mice, A549-Scramble tumors in HFD-fed mice were poorly differentiated, more aggressive and contained highly heterogeneous cell types (spindle cells, cells with high nuclear-cytoplasm ratio, and mitotic cells). Whereas, A549-ShCD36 tumors in NCD-fed mice and even in HFD-fed mice retained well-differentiated glandular structures with more loosely arranged cells, when compared with A549-Scramble in NCD-fed and HFD-fed mice (Fig. 7C). In most of A549-Scramble tumor cells in NCD-fed mice, actin filaments were well organized and distributed evenly throughout the cells. This situation was similar as A549-ShCD36 tumor cells in NCD-fed mice (Fig. 7D). In HFD-fed mice, the actin filaments of A549-Scramble tumor cells formed filopodia, one of the cell motile structures, at the leading edge of the cells, which nearly disappeared in A549-ShCD36 tumor cells in HFD-fed mice (Fig. 7D). The distribution of Arp2 and Arp3 was increased at the root of the formed actin protrusions and partially colocalized with actin-remodeling in A549-Scramble tumor cells in HFD-fed mice (Figs. 7E, S5).

After removing of subcutaneous tumors, the mice were then kept feeding and sacrificed 12 weeks later to investigate oncogenic effect of CD36 on LUAD cell metastasis. Results showed that A549-Scramble cells in HFD-fed mice exhibited a significantly increased metastatic ability to the lung (*p* < 0.05 vs. Scramble NCD), while CD36 knockdown decreased LUAD cell metastasis in HFD-fed groups (*p* < 0.05 vs. Scramble HFD) (Fig. 7F). Pathologic analysis revealed that nodules formed by A549-Scramble cell metastasis to the lung of NCD-fed mice was in neoplasia stage, and nodules formed by A549-Scramble cell metastasis to the lung of HFD-fed mice showed well developed LUAD tumors. A549-ShCD36 cells xenografted in NCD-fed mice did not show obvious metastatic nodules in the lung, and nodules formed by A549-ShCD36 cell metastasis to the lung of HFD-fed mice were still in neoplasia stage (Fig. 7G). The corresponding IHC staining results showed that CD36 expression level was higher in the neoplasia cells, and especially much higher in LUAD tumors, when compared with that in the normal lung cells of ShCD36 NCD-fed mice (Fig. 7H), indicating that higher CD36 expression was accompanied with the development of the metastatic tumor. We further investigated the role of CD36 in regulating lipid and glucose utilization in LUAD cell. When CD36 was inhibited with SSO or its specific shRNA, the uptake of fatty acid (FA) and glucose decreased significantly. Consistent with uptake of the energy substrates, the production of lactate and ATP was proportionally reduced (Fig. S6). The results indicated that: 1) CD36 not only controlled the uptake of fatty acid but also regulated the uptake of glucose as well; 2) the reduced production of lactate and ATP was due to the reduced glucose uptake. Since the intake of glucose and fatty acid decreased, the ability of the cancer cell to maintain viability and build new biomass was restricted. Thus, CD36 shRNA or chemical inhibitor SSO directly inhibited the lung adenocarcinoma cell proliferation and actin remodeling-involved metastasis.

### CD36 played the key role to promote metastatic properties of LUAD cells in HFD-fed nude mice and human LUAD patients

**Fig. 8:**
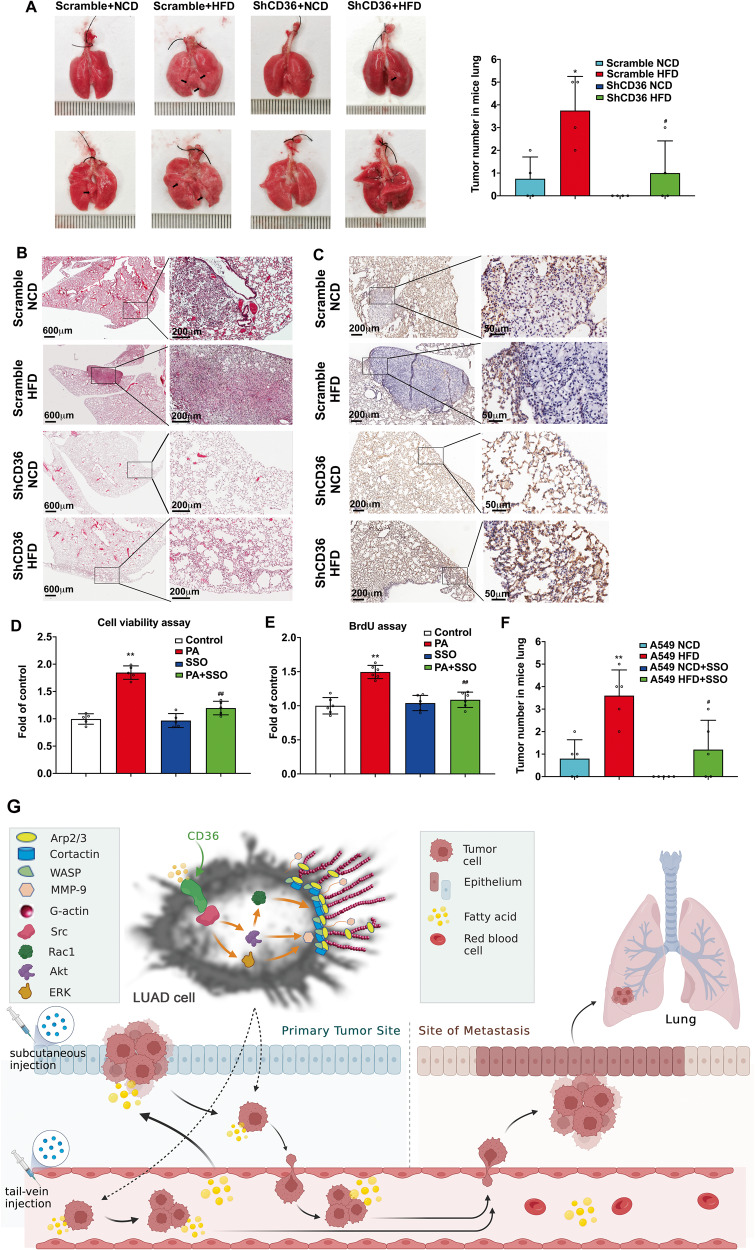
Both CD36 knockdown in LUAD cells and CD36 inhibitor SSO inhibited LUAD cell lung metastasis. **A** Detection of lung metastasis of tail-vein injected LUAD cells. The nude mice fed with NCD and HFD were injected with A549-Scramble or A549-ShCD36 cells through tail-vein. After 12 weeks, the lungs of nude mice were collected and lung metastatic nodules were calculated. Lung surface nodules were indicated as black arrows. Data were mean ± SD, *p < 0.05 vs. Scramble NCD, ^#^*p* < 0.05 vs. Scramble HFD, *n* = 4. **B** Pathologic analyses of the lungs from tail-vein injected LUAD cell mice. Lung tissue sections from mice of A549-Scramble NCD, A549-Scramble HFD, A549-ShCD36 NCD, or A549-ShCD36 HFD were stained with H&E and examined. **C** IHC staining of the lungs from tail-vein injected LUAD cell mice. Lung tissue sections from mice of A549-Scramble NCD, A549-Scramble HFD, A549-ShCD36 NCD, or A549-ShCD36 HFD were stained with CD36 and examined. **D** SSO inhibited PA-enhanced LUAD cell viability. A549 cells were treated with or without 10 μM PA for 48 h, or cells were pretreated with 50 μM SSO for 30 min, which was followed by 10 μM PA treatment or not for another 48 h. MTT assay were performed and non-treatment condition was set up as 1, **p < 0.01 vs. control, ^##^*p* < 0.01 vs. PA + SSO, *n* = 5. **E** SSO inhibited PA-induced LUAD cell proliferation. A549 cells were treated with or without 10 μM PA for 6 h, or cells were pretreated with 50 μM SSO for 30 min, which was followed by 10 μM PA treatment for another 6 h. BrdU assay were performed and non-treatment 6 h condition was set up as 1, **p < 0.01 vs. control, ^##^*p* < 0.01 vs. PA + SSO, *n* = 6. **F** SSO treatment inhibited LUAD cell metastasis in vivo. A549 cells were treated with or without 50 μM SSO for 24 h. Then the cells were injected into nude mice fed with NCD and HFD respectively through tail-vein. The mice were killed after 12 weeks. The lung metastatic nodules were calculated. Data were mean ± SD, **p < 0.01 vs. NCD, ^#^*p* < 0.05 vs. HFD, *n* = 5. **G** Schematic mechanism illustrating the high fat-induced LUAD cells metastasis. HFD leads to increased levels of fatty acids in the body. Upon binding with fatty acid, the CD36 molecules located on plasma membrane function as the initiator of CD36-Src-Akt/ERK signaling, which in turn activates Rac1 via Akt, and up-regulates MMP-9 expression via Akt and ERK. All of these contribute to the fatty acid-induced redistribution of Arp2/3, MMP-9 and cortactin, leading to actin remodeling-involved LUAD cell pulmonary metastasis in high-fat environment.

To further investigate the oncogenic effect of CD36 on LUAD cell metastasis, the A549-Scramble and A549-ShCD36 cells were inoculated into nude mice fed with NCD or HFD via tail-vein injection, respectively. The mice were sacrificed 12 weeks later to examine metastatic nodules in lung, liver and spleen. A549-Scramble cells in HFD-fed mice exhibited a significantly increased metastatic ability to the lung (*p* < 0.05 vs. Scramble NCD), while A549-ShCD36 cells decreased in the total metastatic nodule number in the lung of HFD-fed mice (*p* < 0.05 vs. Scramble HFD) (Fig. 8A). HE staining results revealed that A549-Scramble tumor in lung of NCD-fed mice was smaller with more loosely arranged cells, while A549-Scramble nodules in lung of HFD-fed mice had already developed into larger mature LUAD tumors. Lung nodules derived from A549-ShCD36 cell tail-vein injection in HFD-fed mice were still in neoplasia stage, while no nodules could be seen in NCD-fed mice (Fig. 8B). Consistence with the in situ tumorigenic lung metastasis model (Fig. 7H), when compared CD36 expression with that in the normal lung cells of ShCD36-NCD mice, CD36 expression level was higher in the loosely arranged A549-Scramble tumor cells in lungs of NCD-fed mice, in A549-Scramble tumor marginal cells that were invading the surroundings in lungs of HFD-fed mice, and in A549-ShCD36 neoplasia cells in lungs of HFD-fed mice (Fig. 8C), indicating that CD36 high expression was accompanied with the development of the metastatic tumor. It was worth mentioning that CD36 expression in the mature and closely aligned tumor cells located inside the tumor body, that is, those cancer cells most probably under necrosis in the tumor, was significantly reduced (Fig. 8C). We didn’t find metastatic nodules in the liver and spleen of nude mice (data not shown). These results demonstrated that 1) LUAD tumor cells of tail-vein injection metastasized faster than those injected in situ and developed into mature lung tumors earlier; 2) CD36 exhibited its role in promoting the metastasis of LUAD cells, especially in lipid-oversupply condition.

Furthermore, we performed IHC staining for CD36 and vimentin expression on protein level in human LUAD samples. It is clearly known that vimentin is the well-characterized biomarker of metastasis during lung carcinogenesis [36]. The correlation of CD36 and LUAD metastasis is analyzed. Results showed that the high CD36 level was significantly associated with metastasis status of LUAD patients (*p* = 0.0188) (Fig. S7A and Table S1). Further, the high expressions of CD36 and vimentin were positive-related in LUAD tumors (*p* = 0.0144) (Fig. S7B and Table S1). Thus, high CD36 expression was positive correlation with metastasis in human LUAD.

To further evaluate the therapeutic effect of targeting CD36 on LUAD cell metastasis, SSO, an irreversible chemical inhibitor of CD36, was employed. A549 and SSO-treated A549 cells were inoculated into nude mice fed with NCD or HFD via tail-vein injection, respectively. As shown in Fig. 8D, E, SSO did not produce cell cytotoxicity and significantly inhibited the PA-induced LUAD cell proliferation. Furthermore, inhibition of CD36 via SSO significantly decreased the formation of metastatic LUAD tumors on the lung in HFD-fed mice (Fig. 8F), indicating the CD36 inhibitor could be a potential drug for LUAD treatment.

## Discussion

Our results showed that lipid oversupply promoted the formation and metastasis of LUAD tumors in a CD36-Src-Akt/ERK signal dependent manner (Figs. 7,8). PA stimulation promoted LUAD cell proliferation, migration and invasion, and CD36 was indispensable in these processes (Fig. 1). Though we cannot ignore that CD36-mediated FA uptake promotes the FAs metabolism to provide energy for tumor cell proliferation and metastasis [37], it is more likely that CD36 can induce signal transduction in cells as a cell membrane protein. In our study, upon PA stimulation, CD36 translocated from cytoplasm to LUAD cell membrane, and interacted with Src kinase followed by Src activation. Akt and ERK, locating downstream of Src, were then activated (Fig. 2 and Fig. 3). Consistence with our finding, there is report that CD36 can promote the growth of cervical cancer by activating Src and ERK signaling pathways [11]. Also, CD36 is a key mediator of exogenous FA-induced gastric cancer metastasis [10]. Further, activation of Akt and ERK1/2 signal transduction pathways regulates tumor-associated FASN gene expression, which confers growth and survival advantages in various tumors [38]. It is worth mentioning that both Palmitate (PA) [10, 19] and oleate (OA) could promote cancer cell metastasis [11]. Furthermore, our previous study indicates that the signal transduction induced by OA is similar to PA via CD36. It should be noted that PA treatment impairs insulin sensitivity but OA does not [24]. Moreover, in our in vivo study, the main difference between high-fat diet (HFD) and normal-chew diet (NCD) was the lard addition that accounted for 20% of energy uptake. It has been well documented that PA (16:0) and OA (18:1) are the two main components of lard [24, 39]. Our in vivo results here show that HFD promotes LUAD cell proliferation and metastasis in a CD36-dependent manner. Hence, we select PA to treat LUAD cells in our in vitro experiment.

Approximately 70% of lung cancer patients have advanced or metastatic disease that leads to poor prognosis and low 5-year survival rates [40]. Tumor metastasis occurs via a series of steps including detachment from the primary tumor, migration and invasion into surrounding tissue, intravasation to blood circulation, and finally extravasation to form new tumor colonies at secondary sites [41]. In recent years, attentions have been given to Rac1-GTP enzymes in regulating the reorganization of cytoskeleton proteins, which contributes to the malignant transformation, adhesion, migration and invasion [42–44]. We presented here that Rac1 activity but not RhoA or Cdc42 was increased significantly by PA treatment through CD36-Src-Akt signaling pathway, suggesting that Rac1 participated in triggering actin-remodeling and promoting LUAD tumor metastasis (Fig. 5). The finding is in line with that Rac1 overexpresses in various tumors is closely associated with tumor migration, invasion and poor prognosis of carcinomas [45]. Functional activities of Rac1 correlating with the enhanced activation of Akt and GSK3β play an important role in lung cancer metastasis [46]. Down-regulated Rac1 expression or loss of its function significantly suppressed cancer cell proliferation and metastasis [47]. Furthermore, our results showed that Arp2 and Arp3 were involved in actin remodeling formation, in which Rac1 was upstream of Arp2/3 (Figs. 6A, B and S4A). MMP-9 induced by PA in LUAD cells was in a CD36-Src-ERK/Akt dependent manner and mainly located at the tips of the actin protrusions on cell surface (Fig. 6C, D). Cortactin and N-WASP have been reported to be regulated by Src kinase [48]. IF staining showed the distribution of cortactin and N-WASP changed under PA stimulation, as cortactin translocated to the cell membrane and gathered at the root of actin protrusions (Fig. 6E), while some N-WASP proteins colocalized with actin (Fig. S4). Thus, PA activated Rac1 through CD36-Src-Akt signaling pathway, regulated MMP-9 expression through CD36-Src-Akt/ERK signaling pathway, and then changed the distribution of tubulin, N-WASP, cortactin and Arp2/3 in LUAD cells to trigger actin structural remodeling, and finally promote tumor cells metastasis.

A growing body of evidence suggests that combating cancer metastasis by inhibiting migration and invasion is the key to successful cancer treatment [49, 50]. Our results indicated that targeting inhibition or down-regulation of CD36 in LUAD cells significantly reduced tumor cell proliferation and metastasis, in which Src-Akt/Erk signaling and Rac1 activation-induced actin remodeling were involved. Consistent with ours, Pascual et al. reports that the use of neutralizing antibodies to block CD36 causes almost complete inhibition of metastasis in mouse models of human oral cancer [19]. The therapeutic strategy on targeting CD36 and CD36-Src-Akt/ERK signaling may have effective inhibitory effects on LUAD tumor growth and metastasis.

In conclusion, CD36 functions as the initiator of CD36-Src-Akt/ERK signaling pathway in high-fat environment, which activates Rac1 and finally leads to actin remodeling-involved LUAD cell metastasis (Fig. 8G). These findings provide valuable targets for prevention and treatment of LUAD.

## Materials and methods

### Cell culture, plasmids transfection, and establishment of stable cell lines

Lung cancer cell lines NCI-H23 and A549 were purchased from the American Type Culture Collection. And the cell lines were recently authenticated by STR profiling (The Beijing Genomics Institute, Beijing, China). Lentivirus vectors containing one scramble and three shRNAs for CD36 were purchased from Origene Biotechnology Co., Ltd (Rockville, MD). The CD36 knockdown effect of the three shRNAs were tested and the shRNA (5′-GGACCATTGGTGATGAGAAGGCAAACATG-3′) given the best inhibition effect was selected. Then the plasmids (pLent-U6-GFP-Puro) containing the selected CD36 shRNA and scramble-shRNA, or empty vector (pLent-EF1a-FH-CMV-GFP-p2A-Puro) and the CD36-Flag recombinant plasmid were packed into lentivirus respectively by Weizhen Biotechnology Co., Ltd (Shandong, China). NCI-H23 and A549 cells were cultured in DMEM supplemented with 10% inactivated FBS (Invitrogen, Carlsbad, CA) and transfected with lentivirus respectively. Then the transfected cells were selected with 1 μg/ml puromycin to produce stable transfected cells (designated as NCI-H23-ShCD36, A549-ShCD36, NCI-H23-Scramble, and A549-Scramble, NCI-H23-CD36OE, A549-CD36OE, NCI-H23-Vector, and A549-Vector respectively). Knockdown or overexpressed efficiency was confirmed at protein levels. For transient transfection, plasmids with HA-tagged wide type Rac1 (Rac1 WT), dominant negative Rac1 (Rac1 DN) and constitutively active Rac1 (Rac1 CA) (UMR cDNA Resource Center, Rolla, MO) were transfected into the cells with lipo3000 according to the instructions. Then the lipo-transfected cells were used after 48 h of transfection.

### Assessment of cell viability and cell proliferation

Cell viability and cell proliferation were detected by MTT assay using MTS kit (Progema, Beijing) and Cell Proliferation ELISA, BrdU (colorimetric) kit (Roche, Mannheim, Germany) respectively, according to the manufacturer’s instructions.

### Wound healing and transwell assay

Cells under different treatments were incubated with 10 μg/ml mitomycin-C (Sigma, MO) for 2 h and then starved in serum-free medium for 24 h to suppress proliferation. Then cells were used for wound healing or transwell assay as described [36]. The scratched monolayers or migrated cells in randomly selected fields were observed by light microscopy (Olympus, Japan) and analyzed by Image J software.

### Cell fractionation

Surface protein (Plasma membrane protein), and cytosol protein were extracted for Western blotting according to our previous publication [30]. The protein concentration of these subcellular fractions was determined using the Bio-Rad protein assay.

### Western-blot (WB) and co-immunoprecipitation (Co-IP)

WB and Co-IP were performed as described [24]. WB antibodies against CD36 (ab133625) were purchased from Abcam (Cambridge, MA), antibodies against Src (A19119), Rac1 (A5539), Cdc42 (A1188) and RhoA (A15641) were from Abclonal (Wuhan, China), antibodies against p-Src-Tyr416 (6943S), p-Src-Tyr527 (2105S), p-Akt473 (9271S), Akt (9272S), p-ERK1/2 (4370S), ERK1/2 (4695S), MMP-9 (13667S), GAPDH (5174S), anti-mouse (7076) and anti-rabbit (7074) HRP-linked IgGs, were purchased from Cell Signaling (Boston, MA). Rac1 activation Kit (80501), RhoA activation Kit (80601) and Cdc42 activation Kit (80701) were purchased from NewEast Biosciences (Malvern, PA). IP antibodies against CD36 (sc-7309) and Src (sc-5266) were from Santa Cruz (Dallas, TX).

### Immunofluorescence (IF), immunohistochemistry (IHC), and haematoxylin-eosin (H&E) staining

After proper treatments, cells were fixed with 3% paraformaldehyde in PBS. The tissues in formalin-fixed paraffin sections were sliced to 5 μm thickness for staining. IF and IHC staining were performed as described previously [36]. Primary antibodies of CD36 (A14714), Rac1 (A5539), Cdc42 (A1188) and RhoA (A15641) were from Abclonal (Wuhan, China), Na/K-ATPase (ab32087), Arp2 (ab47654), Arp3 (ab49671) were from Abcam (Cambridge, MA), MMP-9 (13667S), α-tublin (2144S), cortactin (3503S), N-WASP (4848S), HA (3724S) were purchased from Cell Signaling (Boston, MA), Fluor 546 goat anti-rabbit (A11035)/mouse (A11003), Fluor 488 goat anti-rabbit (A11034)/mouse (A11001), Alexa Fluro 546 Phalloidin (A22283) were purchased from Invitrogen (Waltham, MA). CD36 antibody (PAB12463) for IHC was purchased from Abnova (Taibei, TW). Vimentin antibody (46173) was purchased from Cell Signaling (Boston, MA). Protein expression levels were scored as previous description [51, 52]. 5 μm paraffin sections of the tissues were stained with haematoxylin-eosin for 3–5 min. The stained cells were examined using the Leica microscopy (Aperio CS2, Vista, CA) or the Zeiss confocal microscopy LSM880 (Carl Zeiss, Jena, Germany).

### Xenograft model

All animal experiments were approved by the Animal Experimentation Ethics Committee of Shenzhen University. The principles of laboratory animal care were followed and the study was carried out in compliance with the ARRIVE guidelines. The nude mice (BALB/c, male, 3-week-old) were fed with NCD or HFD for 1 week. Mice were housed in a temperature-regulated environment (20 °C) with a reversed 12:12 h light–dark cycle. Then the NCD- or HFD-fed mice were transplanted subcutaneously with A549-Scramble or A549-CD36 shRNA cells (designated as A549-ShCD36 NCD, A549-ShCD36 HFD, A549-Scramble NCD, and A549-Scramble HFD, respectively) for tumorigenicity and metastasis assay. Briefly, the designated cells (1 × 10^6^) were S.C. implanted into the left and right dorsal flank of 4-week-old male nude mice (nu/nu, *n* = 10/group, randomized group), respectively. Subcutaneous tumors were measured in two dimensions by external caliper and Tumor volume (*V*) was estimated by formula [length × width (mm)^2^]/2 [36]. The size of tumor was monitored for 5 weeks, then the tumors were collected while the mice were under anesthesia. After suturing, the mice were continued to feed with NCD or HFD for another 12 weeks. The mice were sacrificed by cervical dislocation and tumor metastasis to lung were observed. For further proving lung metastasis formation, 5 × 10^5^ A549-shCD36 and A549-scramble cells were injected into the lateral tail vein of the NCD- or HFD-fed mice respectively (nu/nu, *n* = 4/group, randomized group). Mice were sacrificed 12 weeks later, and the lung, liver and spleen of each mouse were removed and subjected to formaldehyde fixation and followed by IF, H&E and IHC staining. To detect the effect of CD36 inhibitor SSO (sulfosuccinimidyl oleate) on LUAD cell metastasis, 5 × 10^5^ cells (Non-treatment A549 cells and SSO-treated A549 cells) were injected into the lateral tail vein of the NCD- or HFD-fed mice respectively (nu/nu, *n* = 5/group, randomized group). Mice were killed after 12 weeks. The lungs of nude mice were collected and lung metastatic nodules were calculated.

### Analysis of mice lipid profiles

Plasma FFAs (Wako 294-63601, Osaka, Japan), triglyceride (TG) (BioSino 192061, Beijing, China) and total cholesterol (TC) (BioSino 208051, Beijing, China) were measured by enzymatic colorimetric kits according to the products instructions.

### Fatty acid and glucose uptake

Cells were prepared in 12-well plate with 80% confluence in each well. Then cells were treated with 50 μM SSO for 30 min or left untreated. Then supernatant was removed and cells were washed with warmed PBS for two times. For fatty acid assay, 350 μl DMEM with 0.25 mM PA (no serum) was added to each well and the plate was incubated at 37 °C for 40 min. The supernatant was used for FA measurement according to the instructions of the kit (NEFA LabAssay Kit, Cat# 294-63601, FUJIFILM Wako Pure Chemical Corporation, Osaka, Japan). For glucose uptake assay, 350 μl DMEM with 1 mM glucose (no serum) was added to each well and the plate was incubated at 37 °C for 40 min. The supernatant was used for glucose measurement according to the instructions of the kit (GlucoseColorimetric Assay Kit, Cat# E-BC-K234-S, Elabscience Biotechnology Co., Ltd., Wuhan, China). The cells in each well were lysised with RIPA for protein assay. Finally, the absorbance value of fatty acid or glucose was corrected by the amount of protein in each well.

### Lactate and ATP assay

Cells were prepared in 96-well plate with 90% confluence in each well. Then cells were treated with 50 μM SSO for 30 min or left untreated. Then the lactate in the supernatant and the ATP in the cells were measured according to the instructions of the Kit (Glycolysis/OXPHOS Assay Kit, Cat#G270, DOJINDO Laboratories, Kumamoto, Japan).

### Human samples

50-Paired human primary LUAD and adjacent normal lung tissues were collected immediately after surgical resection at the Prince of Wales Hospital (Hong Kong, China). The study was performed in accordance with the ethical principles and guidelines for human research of the Helsinki Declaration, and human ethics approval (2014.649 and 2015.729) was obtained from the joint Chinese University of Hong Kong-New Territories East Cluster Clinical Research Governance and Management Committee. An informed consent for human tissues for research purposes only was obtained from all patients recruited. All tumor and non-tumor tissue specimens were confirmed by histological examination. The specimens were snap-frozen in liquid nitrogen and stored at −80 °C and were also fixed in 10% formalin and embedded for histochemical staining examination.

### Statistical analysis

Statistical analyses were performed using GraphPad Prism, version 9.0 (GraphPad Software). To compare the difference between two groups, independent sample *t* test (two tailed Student’s *t* test) was used. Based on the CD36/vimentin expression levels in tumor tissues and the paired non-tumor tissues, the expression level was graded. When the expression of CD36 and vimentin level in each paired sample was considered, the expression in non-tumor tissue was set up as the normal, and the expression in tumor tissue was graded as normal/high expression in comparison with the non-tumor tissue. The clinic-pathologic features in patients with relative expressing CD36 and vimentin were compared using Pearson’s Chi-squared test or Fisher’s exact test for categorical variables. *p* < 0.05 was considered statistically significant. All experiments were performed at least three times.

## Supplementary information

Supplementary Table and Figures

Original Data File

aj-checklist-CDDis

## Data Availability

All data generated or analyzed during this study are included in this published article and its supplementary information files.
