# Supplementary material for: The activated CD36-Src axis promotes lung adenocarcinoma cell proliferation and actin remodeling-involved metastasis in high-fat environment
Source: Cell Death Dis. 2023 Aug 23;14(8):548. doi: 10.1038/s41419-023-06078-3 (PMC10447533; doi:10.1038/s41419-023-06078-3)
Supplement: Supplementary file 1 — Supplementary Table and Figures [file 41419_2023_6078_MOESM1_ESM.docx]

**Supplementary Information**

**The activated CD36-Src axis** **promotes lung adenocarcinoma cell proliferation and actin remodeling-involved metastasis in high-fat environment**

Li-Zhong Liu^1^, Bowen Wang^1#^, Rui Zhang^2^, Zangshu Wu^2^, Yuxi Huang^1^, Xiaoyang Zhang^1^, Jiaying Zhou^1^, Junbo Yi^1^, Jian Shen^2^, Ming-Yue Li^2, 3^, Ming Dong^2*^

^1^ Department of Physiology, School of Basic Medical Sciences, Shenzhen University Medical School, Shenzhen University, Shenzhen 518055, Guangdong, China.

^2^ GuangZhou National Laboratory, No.9 XingDaoHuanBei Road, Guangzhou International Bio Island, Guangzhou 510005, Guangdong, China.

^3^ Faculty of Medicine, The Chinese University of Hong Kong, Hong Kong, China

^#^ Current Address: Guangdong Medical Academic Exchange Center, Yuexiu District, Guangzhou, Guangdong, China

^*^**Corresponding author**: Ming Dong, GuangZhou National Laboratory, No.9 XingDaoHuanBei Road, Guangzhou International Bio Island, Guangzhou 510005, Guangdong, China. Tel: 18676711598; E-mail: dong_ming@gzlab.ac.cn.

**Contents:**

**Supplementary Figures S1 to S7**

**Supplementary Table S1**

**Figure S1**

**
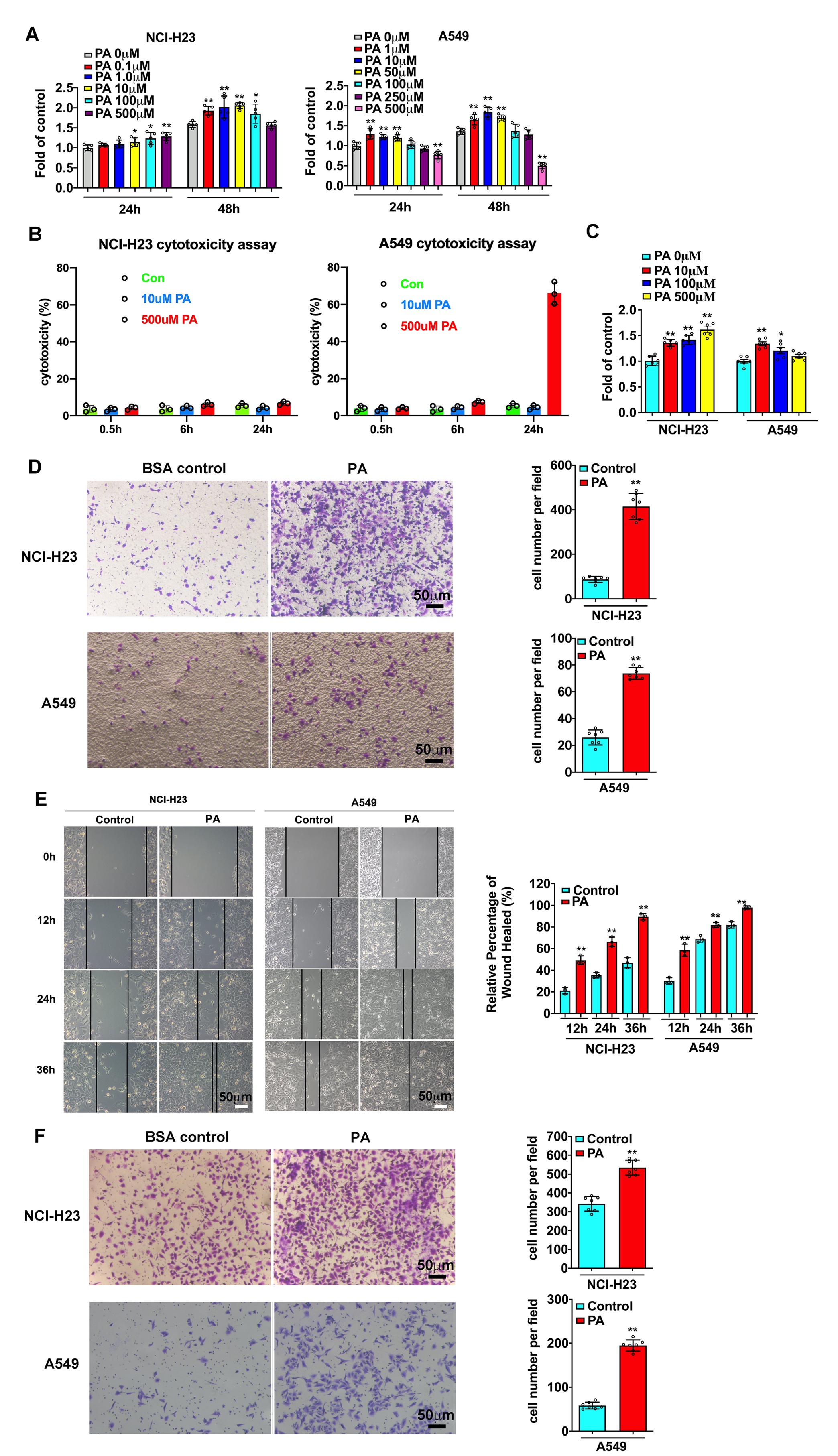
**

**Fig. S1.** **PA promoted LUAD cell proliferation and metastasis. (A) Dose- and time-dependent assay of cell viability.** NCI-H23 and A549 cells treated with the indicated concentration of PA (PA: BSA = 1: 3) for 24 h or 48 h were applied for MTT assay. 0 μM PA (non-treatment) 24 h was set up as 1, **p* <0.05 or ***p* <0.01 vs. 0 μM PA in each time point, n = 3. **(B) PA cytotoxicity assay.** NCI-H23 and A549 cells treated with 10 μM or 500 μM PA (PA: BSA = 1: 3) for different time as indicated were applied for cytotoxicity measurement with LDH Assay kit (ab65393, Abcam, Cambridge, MA). Non-PA treatment was set as control. **(C) PA effect on LUAD cell proliferation.** NCI-H23 and A549 Cells treated with the indicated concentration of PA for 6h were applied for BrdU assay. 0 μM PA (non-treatment) 6 h was set up as control, **p* <0.05, or ***p* <0.01 vs. 0 μM PA, n = 3. **PA promoted LUAD cell (D) migration and (F) invasion - transwell assay.** NCI-H23 or A549 cells treated by 10 μM PA for 24 h were detected by transwell assay for cell migration and invasion respectively. Images were taken using light microscopy (scale bar: 50 μm). The numbers of migration cells in seven randomly selected fields were counted and the average number of cells in one field was calculated and expressed as the mean ± SD, ***p<0.*01 vs control, n = 3. **(E) PA promoted LUAD cell migration-wound healing assay.** NCI-H23 and A549 cells were incubated with 10 μM PA for different periods. Cell migration was detected by wound-healing assay. Images were taken using light microscopy (scale bar: 50 μm). The relative percentage of wound healed was expressed as mean ± SD of three independent experiments, ***p* <0.01 vs. relative control, n = 3.

**Figure S2**

**
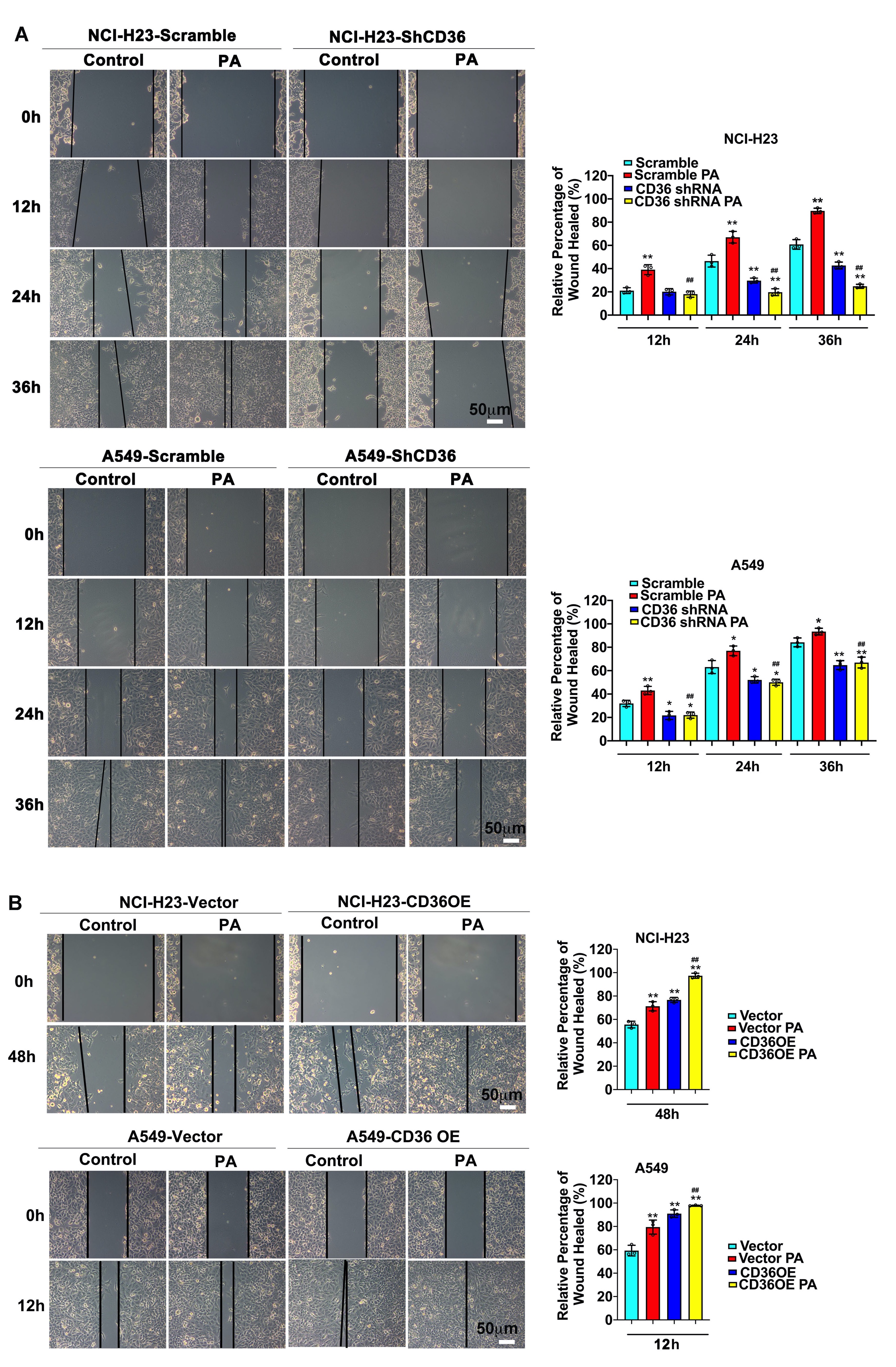
**

**Fig. S2. CD36 was required for PA-induced cell migration.** **(A)** Scramble and ShCD36 cells of NCI-H23 and A549 were incubated with 10 μM PA for different periods. Cell migration was detected by wound-healing assay. Images were taken using phase contrast microscope (Nikon). The relative percentage of wound healed was expressed as mean ± SD of three independent experiments ***p* < 0.01 vs relative Scramble control condition, ^##^*p* < 0.01 vs relative scramble + PA. **(B)** Vector and CD36OE cells of NCI-H23 and A549 were incubated with 10 μM PA for the indicated periods. Cell migration was detected by wound-healing assay. Images were taken using phase contrast microscope (Nikon). The relative percentage of wound healed was expressed as mean ± SD of three independent experiments. **p* < 0.05 and ***p* < 0.01 vs. relative Vector control, ^##^*p* < 0.01 vs. relative Vector + PA.

**Figure S3**

**
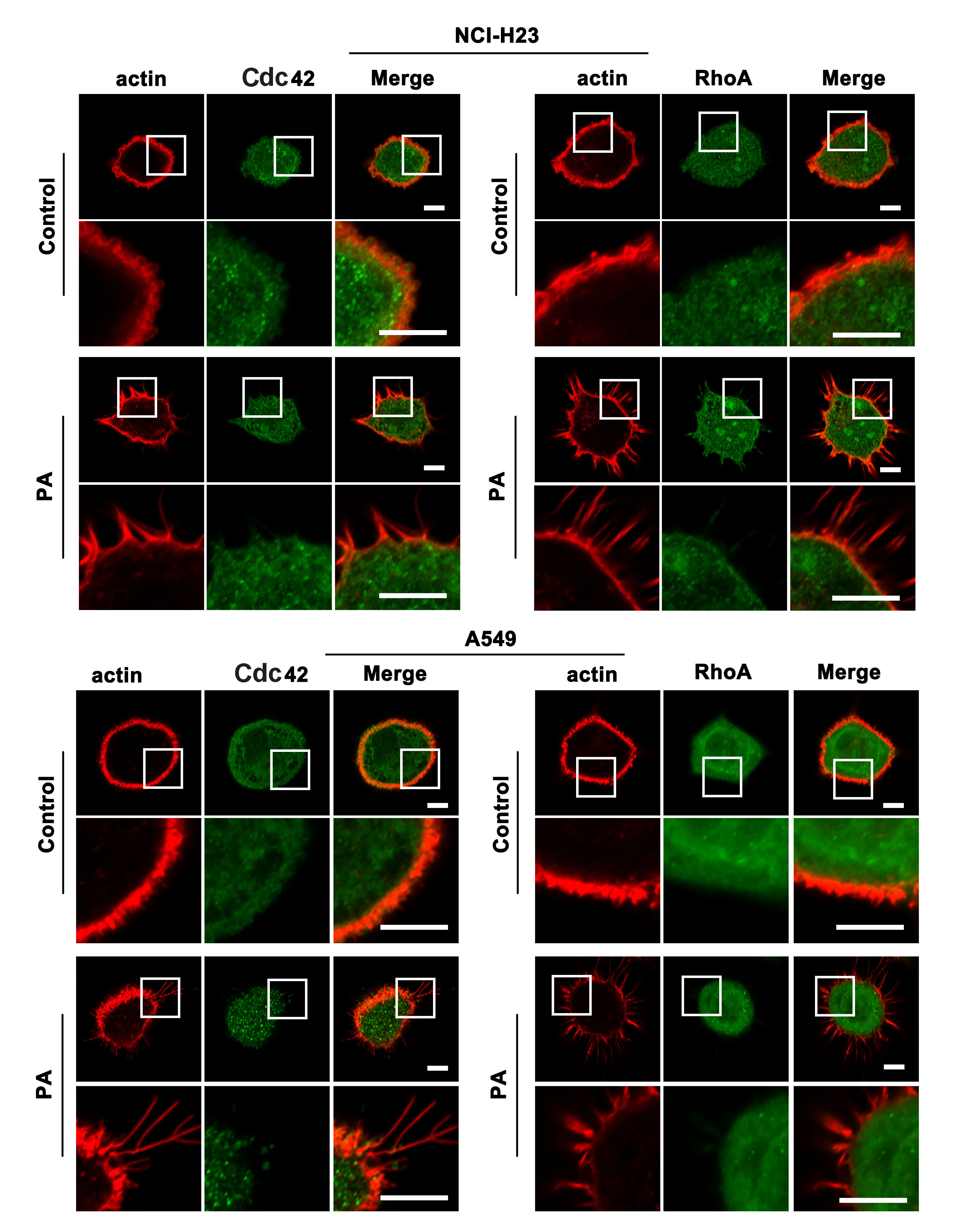
**

**Fig. S3.** **Cdc42 and RhoA showed no colocalization with the finger-like actin remodeling upon PA stimulation.** NCI-H23 and A549 cells were treated with or without 500 μM PA for 0.5 h. Then cells were double stained for actin (red) and Cdc42 or RhoA (green) respectively. Bar, 5 μm. The images were representative of three experiments.

**Figure S4**


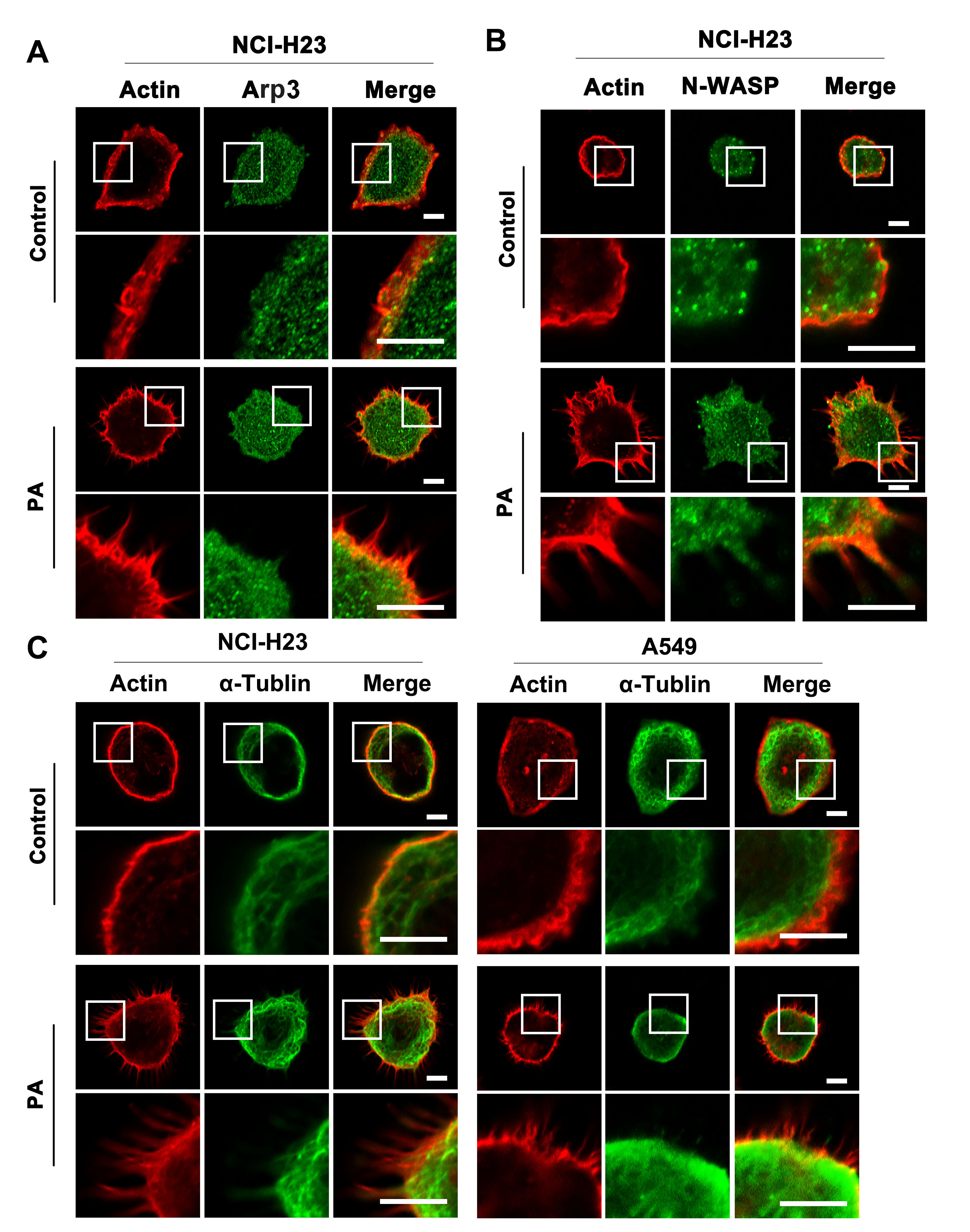


**Fig. S4. PA facilitated redistribution of Arp3/N-WASP/Tublin and their colocalization with actin remodeling. (A-C)** NCI-H23 and A549 cells were treated with or without 500 μM PA for 0.5 h. Cells were double stained for actin (red) and Arp3 (green), N-WASP (green), and α-tublin respectively. Bar, 5 μm. The images were representative of three experiments.

**Figure S5**


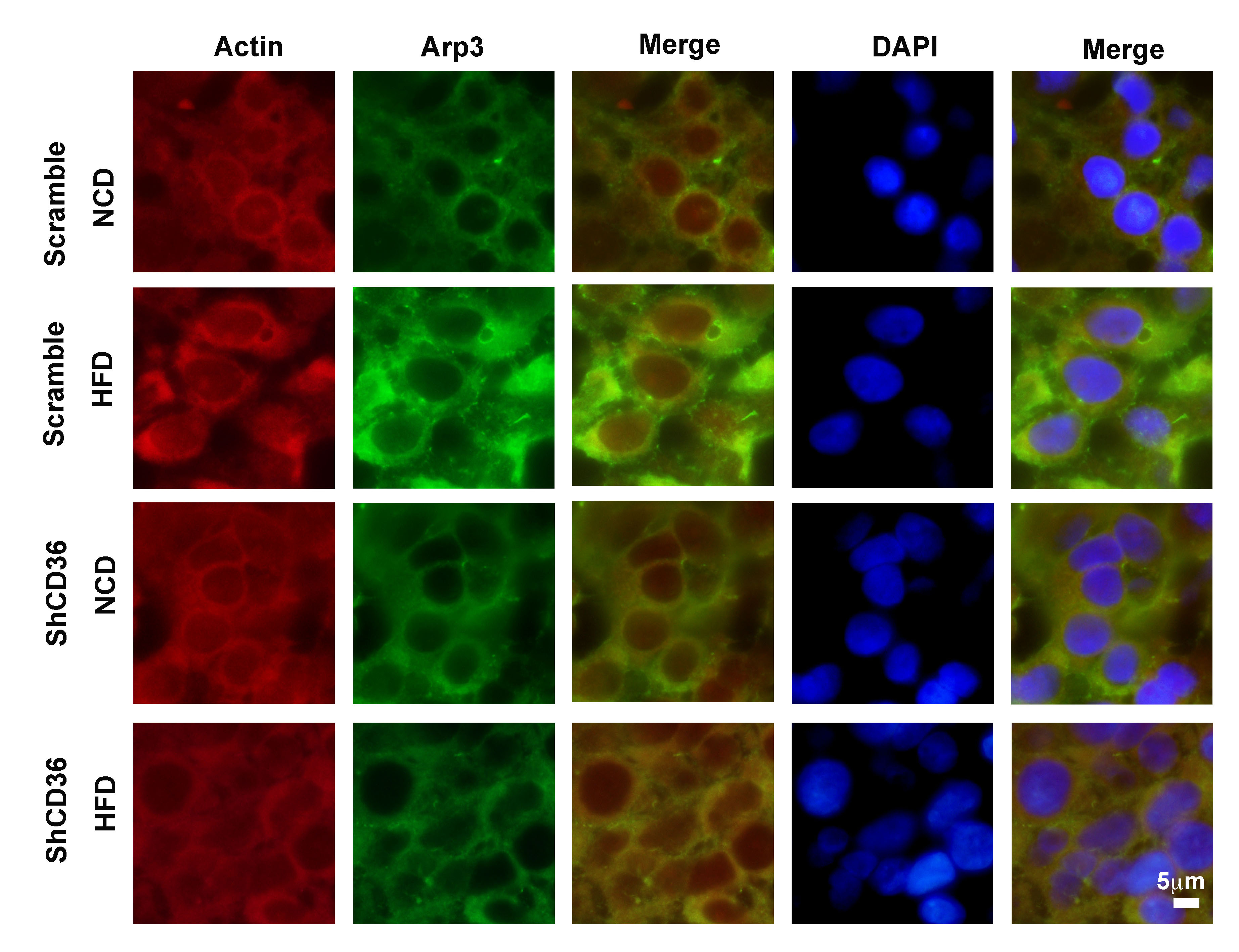


**Fig. S5. CD36 knockdown in LUAD cells inhibited Arp3 accumulation and colocalization with the reformed actin in lung tumor cells of HFD mice.** Tumor tissue cells were double stained for actin (red) and Arp3 (green) respectively. The representative IF staining image of tumors generated from A549-Scramble NCD, A549-Scramble HFD, A549-ShCD36 NCD, or A549-ShCD36 HFD were shown. Bar, 5 μm. The images were representative of three experiments.

**Figure S6**

**
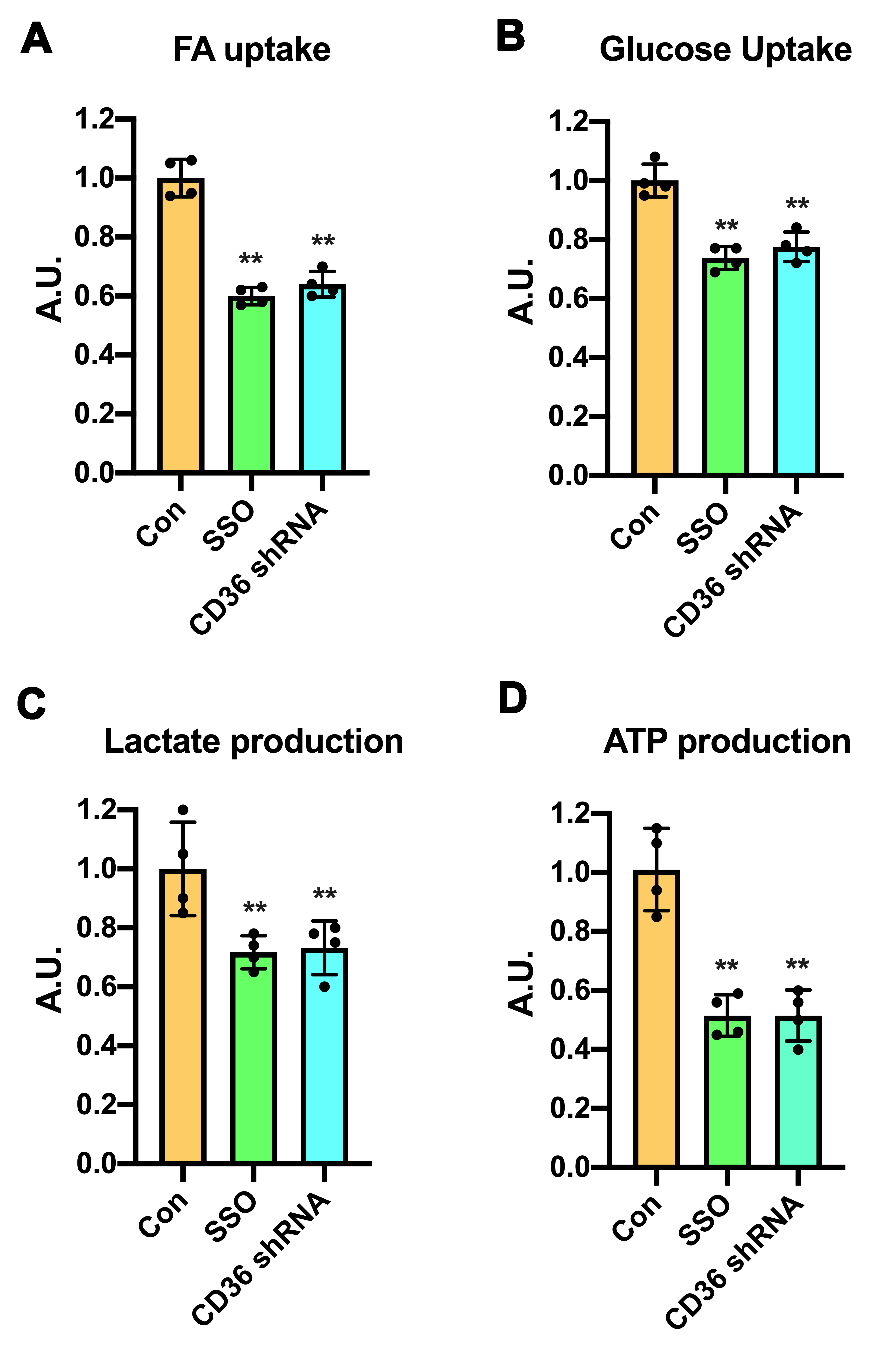
**

**Fig. S6. Effect of CD36 inhibition on fatty acid and glucose utilization. (A) FA uptake assay.** **(B) Glucose uptake assay. (C) Lactate assay. (D) ATP assay.** Con: A549-Scramble. ***p* < 0.01 vs. Con. n = 4.

**Figure S7**

**
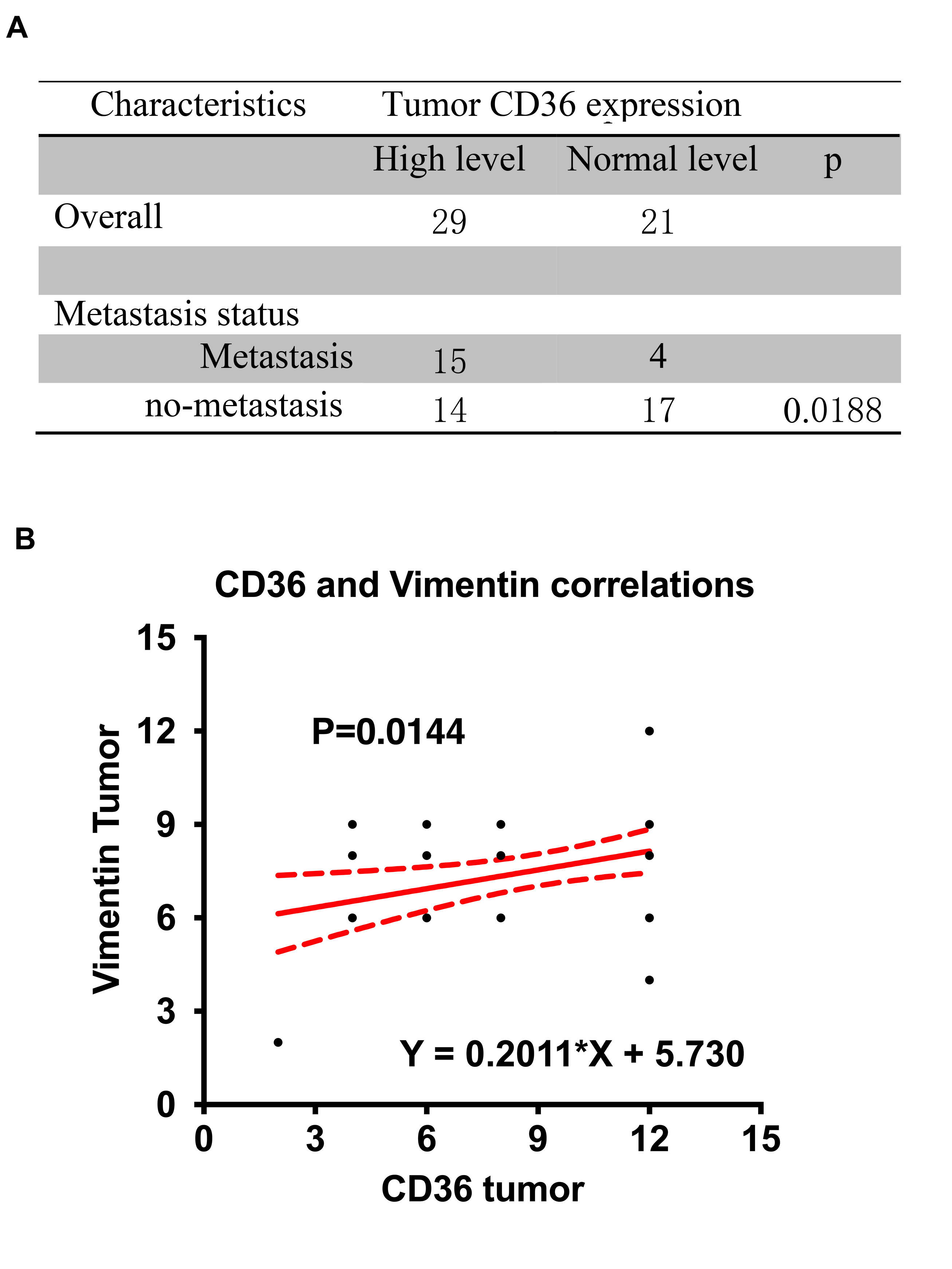
**

**Fig. S7.** **CD36 is positive correlation with metastasis in human LUAD tissues.** (A) Baseline demographic characteristics of 50 human LUAD patients underwent CD36 analysis. The clinic-pathologic features in patients with relative expressing CD36 were compared using Pearson’s chi-squared test for categorical variables. p<0.05 was considered statistically significant. (B) Positive correlation of CD36 and Vimentin in LUAD tumor. The correlation of CD36 and Vimentin expression levels in 50 LUAD tumor tissues were analyzed (p<0.05).

**Table S1. Clinical characteristics of patients and CD36/Vimentin IHC score.**

| **Patient**  **No.** | **sex** | **Pathology type** | **age** | **Pathology stage and remark** | **CD36 score** | | **Vimentin score** | |
| --- | --- | --- | --- | --- | --- | --- | --- | --- |
|  |  |  |  |  | **Tumor** | **Non-tumor** | **Tumor** | **Non-tumor** |
| 1 | M | adenocarcinoma | 71 | Ib; moderately differentiated | 6 | 6 | 8 | 6 |
| 2 | M | adenocarcinoma | 54 | IIb; moderately differentiated, present close to pleura. | 8 | 6 | 8 | 6 |
| 3 | M | adenocarcinoma | 75 | IIb; moderately differentiated, extends to the intercostal soft tissue | 6 | 4 | 6 | 6 |
| 4 | M | adenocarcinoma | 58 | IV; Poorly differentiated, with erosion into large airways. | 12 | 6 | 8 | 6 |
| 5 | M | adenocarcinoma | 64 | Ia; poorly differentiated, vascular permeation | 8 | 4 | 8 | 6 |
| 6 | M | adenocarcinoma | 60 | Ib; poorly differentiated | 12 | 4 | 8 | 6 |
| 7 | M | adenocarcinoma | 61 | Ib; moderately differentiated | 12 | 4 | 6 | 6 |
| 8 | M | adenocarcinoma | 65 | Ia; well differentiated | 12 | 8 | 12 | 8 |
| 9 | M | adenocarcinoma | 42 | IIIa; poorly differentiated; with erosion into chest wall | 12 | 8 | 12 | 8 |
| 10 | M | adenocarcinoma | 71 | IIb; moderately to poorly differentiated; extends to the peribronchial soft tissue | 12 | 6 | 6 | 6 |
| 11 | M | adenocarcinoma | 58 | IIb; moderately differentiated, extends through the pleura to reach the intercostal soft tissue | 6 | 8 | 6 | 8 |
| 12 | M | adenocarcinoma | 75 | IIIb; moderately differentiated | 12 | 12 | 8 | 8 |
| 13 | M | adenocarcinoma | 71 | Ib; poorly differentiated | 6 | 4 | 6 | 6 |
| 14 | M | adenocarcinoma | 59 | IIb; moderately differentiated | 12 | 6 | 8 | 12 |
| 15 | M | adenocarcinoma | 75 | Ib; moderately differentiated | 4 | 6 | 6 | 8 |
| 16 | M | adenocarcinoma | 74 | IIb; well differentiated | 8 | 12 | 8 | 8 |
| 17 | F | adenocarcinoma | 67 | IIa; moderately to poorly differentiated, extends to the pleura | 8 | 6 | 6 | 6 |
| 18 | M | adenocarcinoma | 63 | Ib; moderately differentiated | 6 | 8 | 9 | 9 |
| 19 | M | adenocarcinoma | 88 | Ib; poorly differentiated, vascular permeation | 6 | 2 | 8 | 6 |
| 20 | M | adenocarcinoma | 88 | IIb; moderately differentiated | 4 | 8 | 8 | 12 |
| 21 | M | adenocarcinoma | 75 | IV; moderately differentiated; intrapulmonary metastasis | 12 | 8 | 6 | 8 |
| 22 | M | adenocarcinoma | 71 | Ib; moderately differentiated | 12 | 6 | 4 | 6 |
| 23 | M | adenocarcinoma | 82 | IIIa; moderately differentiated | 4 | 6 | 6 | 6 |
| 24 | M | adenocarcinoma | 62 | IIb; moderately differentiated, extend to pleura | 4 | 4 | 9 | 9 |
| 25 | M | adenocarcinoma | 60 | Ib; moderately differentiated | 4 | 4 | 8 | 6 |
| 26 | M | adenocarcinoma | 80 | Ib; poorly differentiated, tumor cells seen at bronchial margin | 12 | 8 | 8 | 8 |
| 27 | M | adenocarcinoma | 51 | IIb; moderately differentiated | 12 | 12 | 12 | 9 |
| 28 | F | adenocarcinoma | 71 | Ib; moderately differentiated | 12 | 8 | 8 | 8 |
| 29 | M | adenocarcinoma | 77 | IIb; moderately differentiated | 12 | 12 | 8 | 8 |
| 30 | M | adenocarcinoma | 59 | Ib; moderately differentiated, close to pleura, be metastasis | 12 | 8 | 8 | 8 |
| 31 | M | adenocarcinoma | 75 | IIb; poorly differentiated, metastasis | 12 | 4 | 8 | 6 |
| 32 | M | adenocarcinoma | 77 | IIb; moderately differentiated | 12 | 9 | 12 | 9 |
| 33 | M | adenocarcinoma | 81 | IIb; moderately differentiated | 6 | 8 | 6 | 6 |
| 34 | M | adenocarcinoma | 50 | IIb; well differentiated | 8 | 8 | 8 | 6 |
| 35 | M | adenocarcinoma | 75 | IIa; well differentiated | 8 | 6 | 8 | 6 |
| 36 | F | adenocarcinoma | 77 | IIb; moderately differentiated | 2 | 4 | 2 | 2 |
| 37 | F | adenocarcinoma | 49 | IIIb; poorly differentiated, pleural metastasis present | 6 | 4 | 6 | 4 |
| 38 | M | adenocarcinoma | 70 | Ia; moderately differentiated | 8 | 8 | 6 | 6 |
| 39 | F | adenocarcinoma | 85 | IV; poorly differentiated, extends to the pleura | 6 | 4 | 6 | 6 |
| 40 | F | adenocarcinoma | 59 | IIIa; moderately differentiated. | 12 | 8 | 9 | 6 |
| 41 | F | adenocarcinoma | 65 | IIb; moderately differentiated, close to pleura | 8 | 4 | 9 | 6 |
| 42 | F | adenocarcinoma | 53 | Ib; poorly differentiated | 12 | 6 | 6 | 6 |
| 43 | F | adenocarcinoma | 66 | Ia; well differentiated | 6 | 8 | 6 | 6 |
| 44 | M | adenocarcinoma | 75 | IV; moderately differentiated | 12 | 9 | 9 | 9 |
| 45 | F | adenocarcinoma | 68 | IIa; well differentiated | 4 | 8 | 8 | 6 |
| 46 | F | adenocarcinoma | 71 | Ib; moderately differentiated | 12 | 9 | 6 | 6 |
| 47 | F | adenocarcinoma | 61 | IV; well differentiated | 12 | 12 | 6 | 6 |
| 48 | F | adenocarcinoma | 82 | Ia; moderately differentiated, poor pulmonary metastasis | 12 | 12 | 8 | 6 |
| 49 | F | adenocarcinoma | 46 | Ib; moderately differentiated; extend to pleura | 12 | 12 | 8 | 6 |
| 50 | M | adenocarcinoma | 60 | Ia; well differentiated | 12 | 4 | 8 | 6 |
