# Supplementary figures and images for: The activated CD36-Src axis promotes lung adenocarcinoma cell proliferation and actin remodeling-involved metastasis in high-fat environment
Source: Cell Death Dis. 2023 Aug 23;14(8):548. doi: 10.1038/s41419-023-06078-3 (PMC10447533; doi:10.1038/s41419-023-06078-3)

Figure 1A

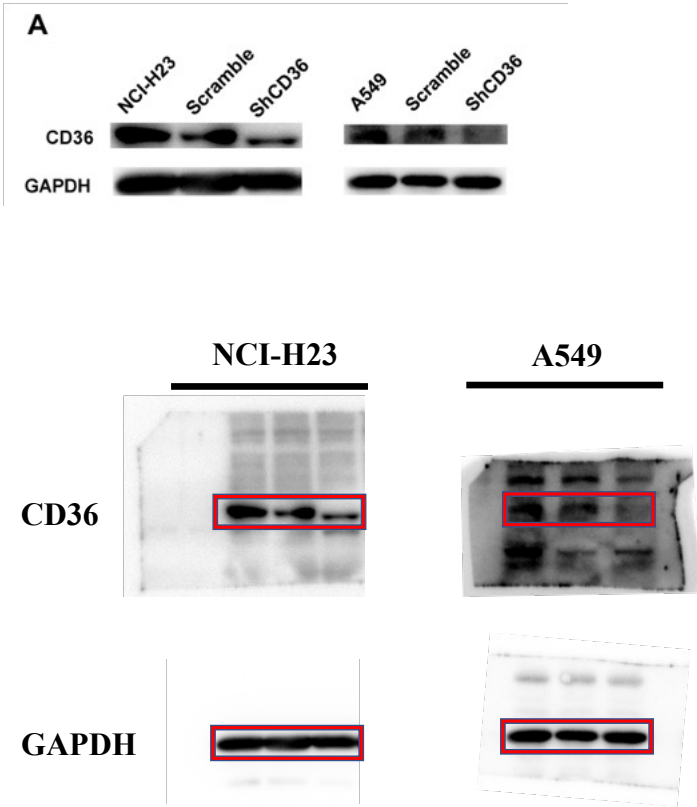

Figure 1B

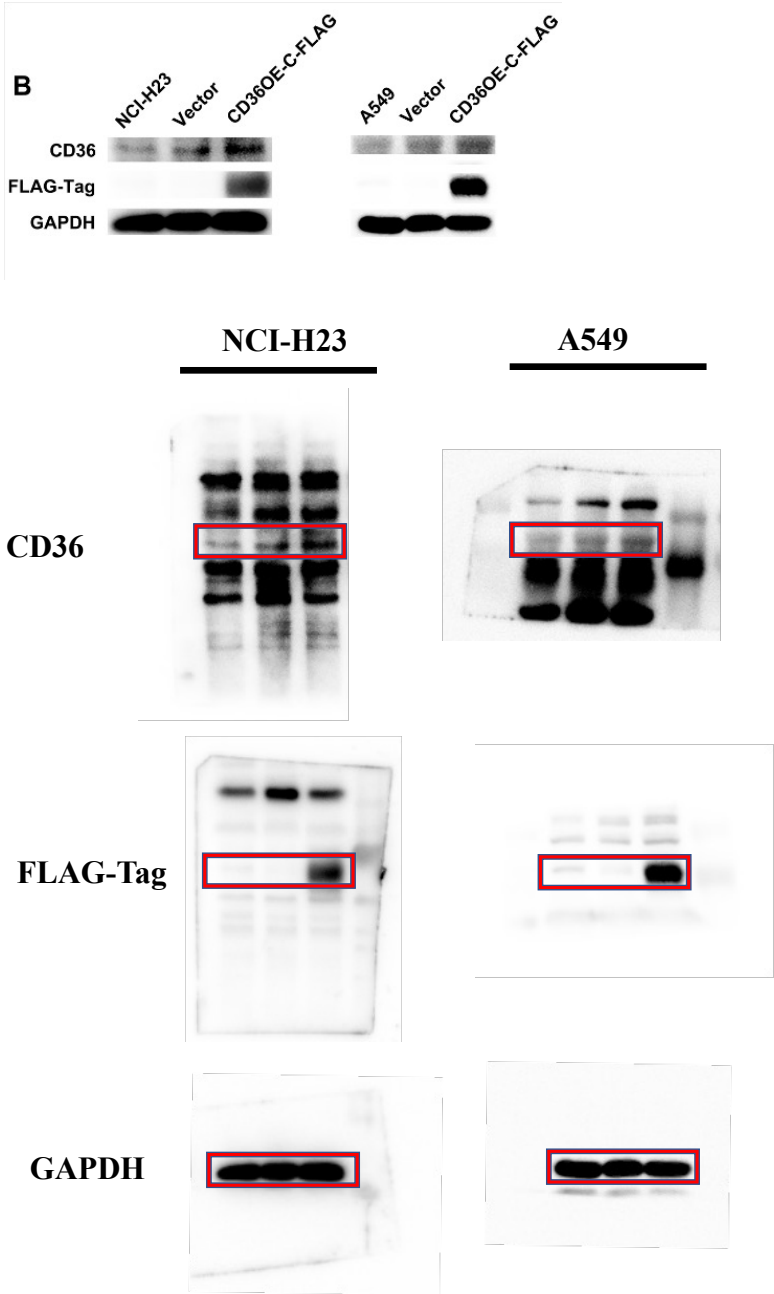

Figure 3D

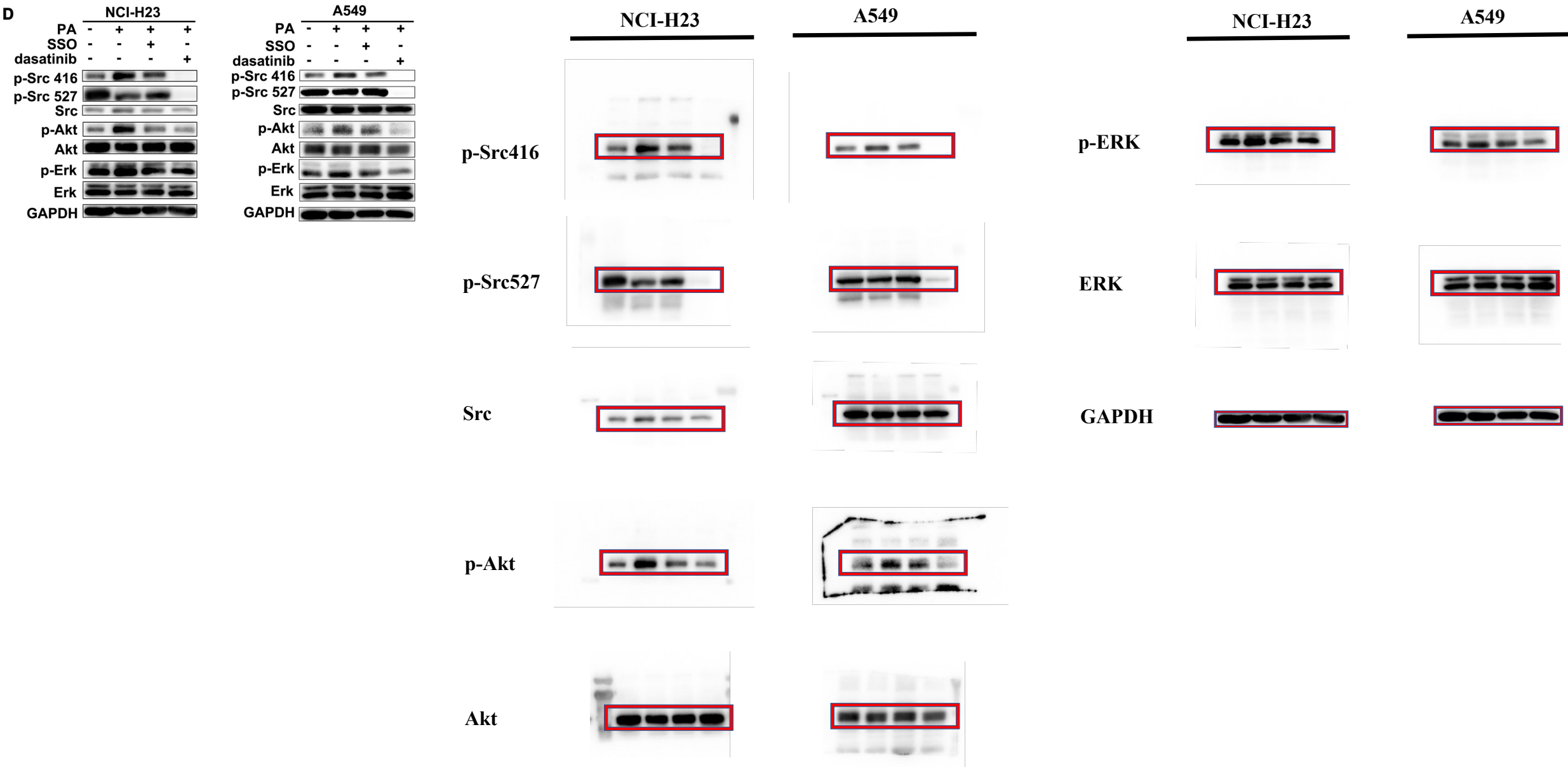

Figure 4C

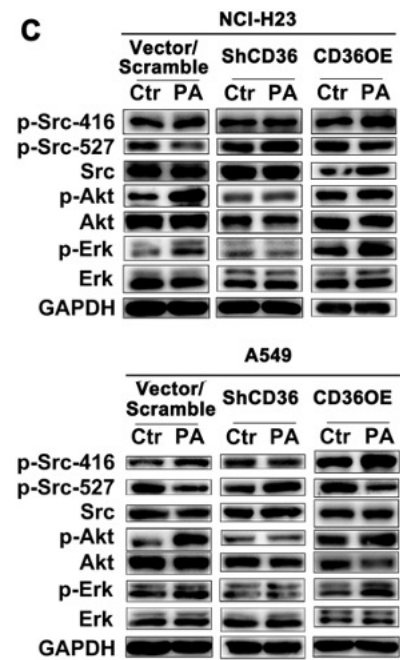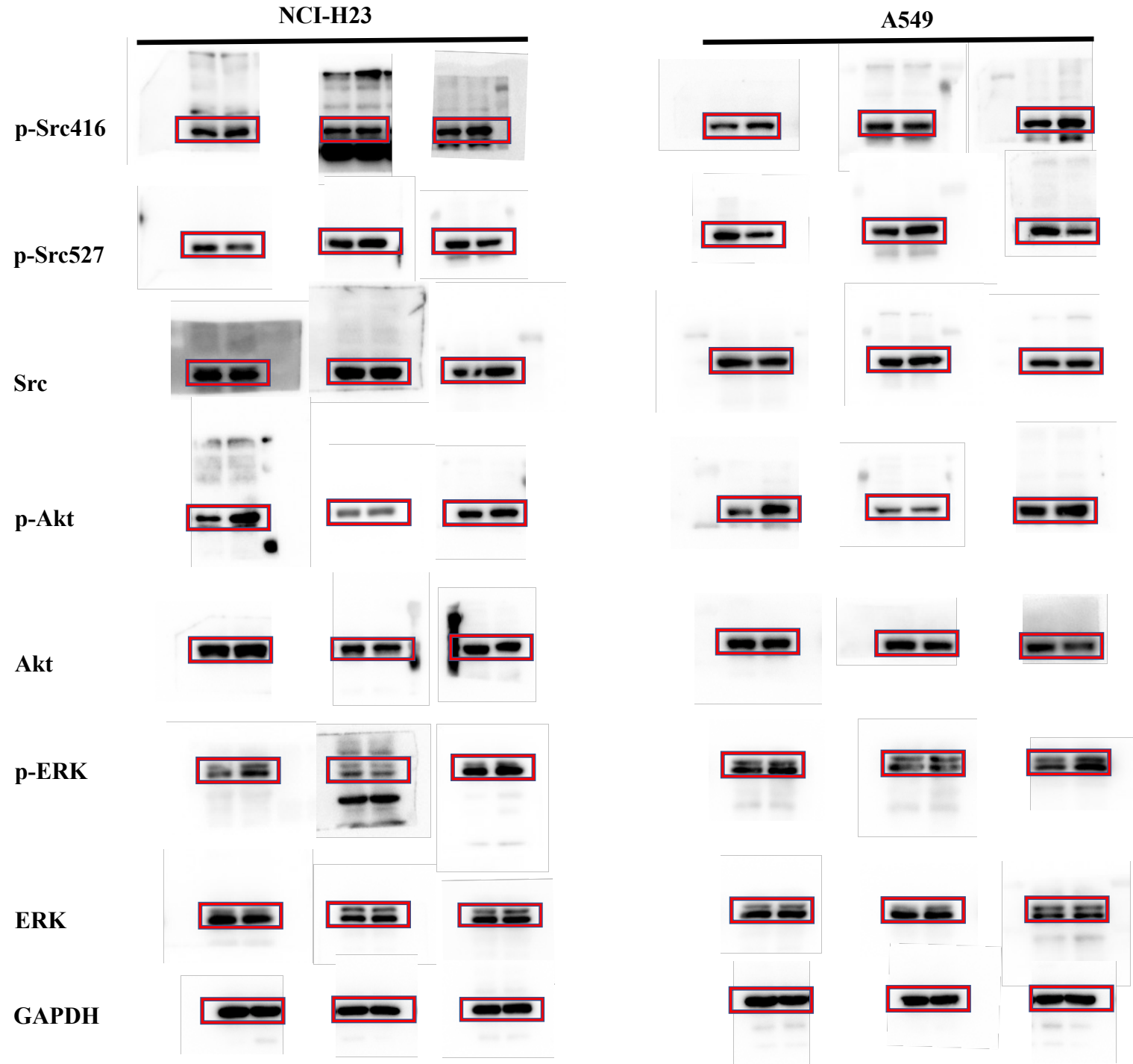

Figure 5A

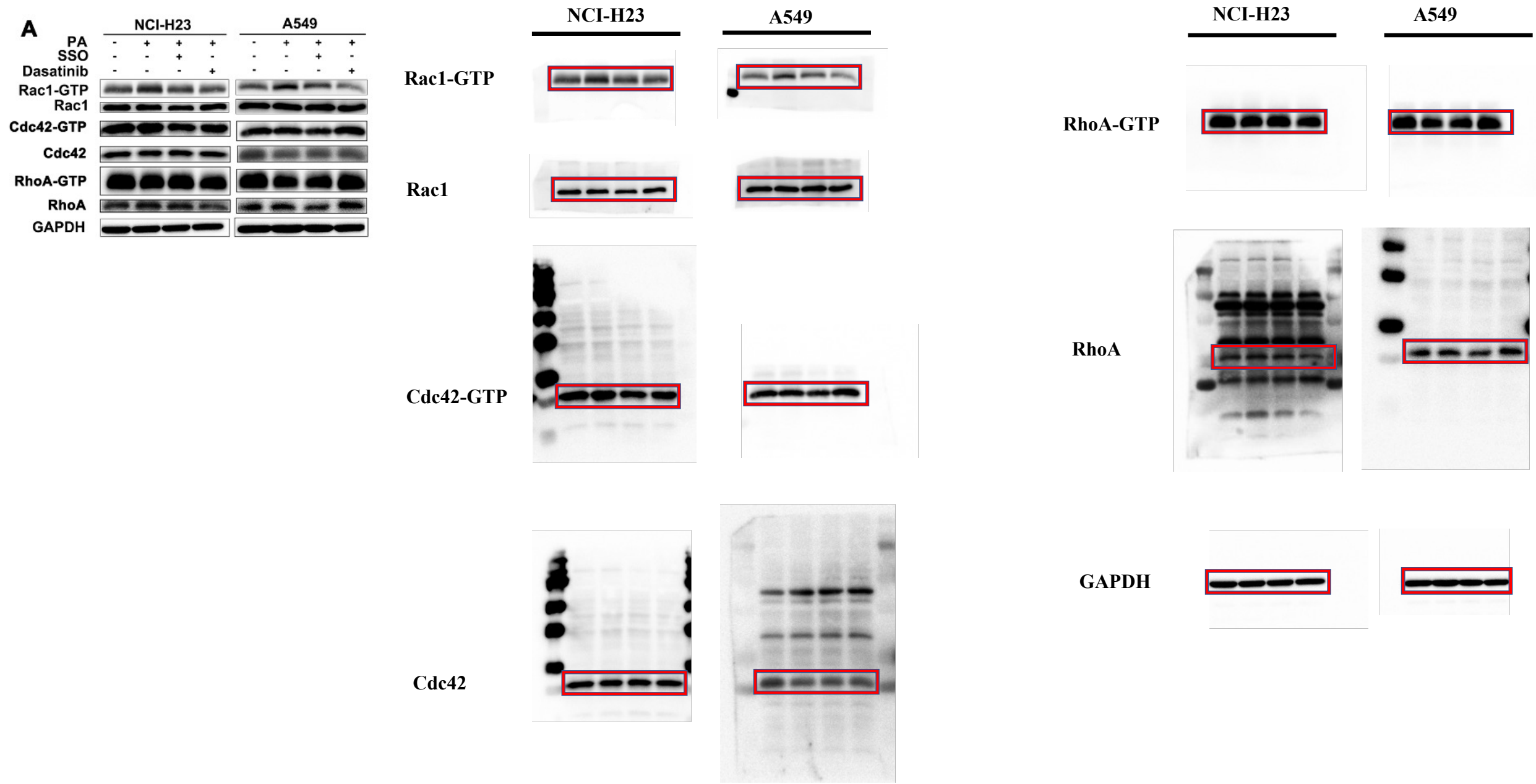

Figure 5B

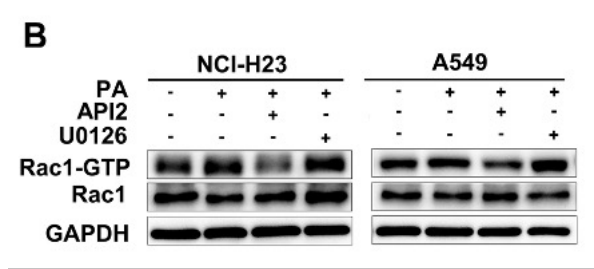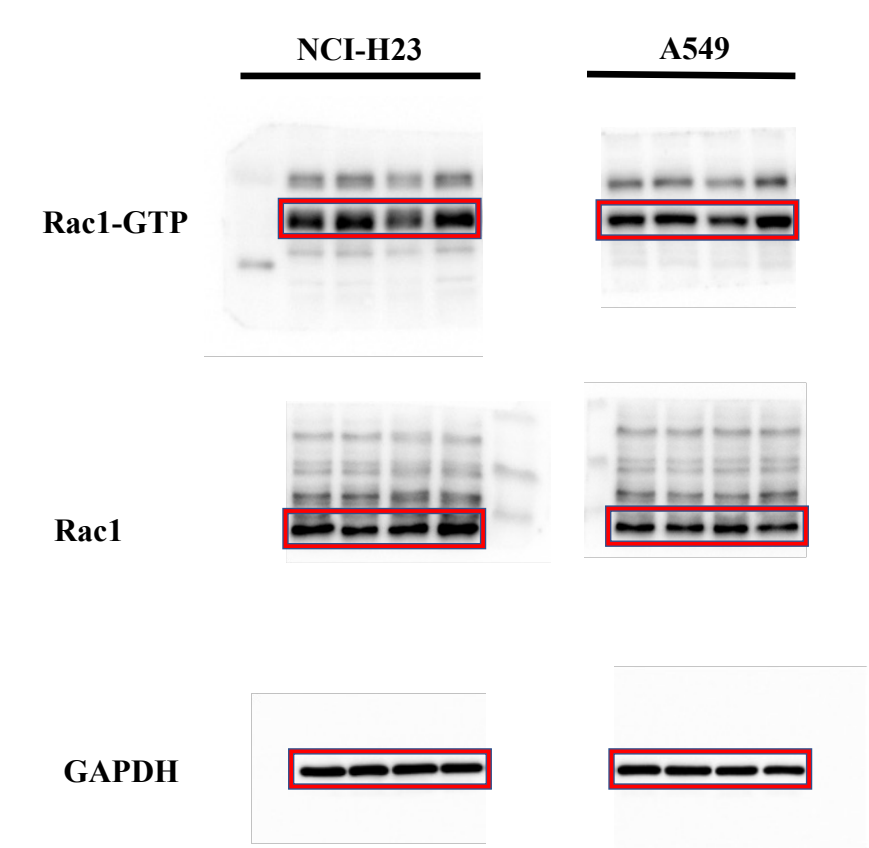

Figure 6C

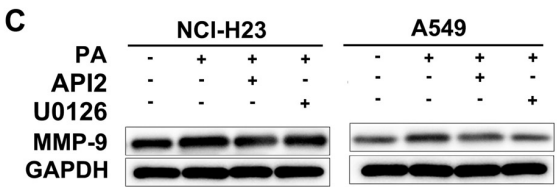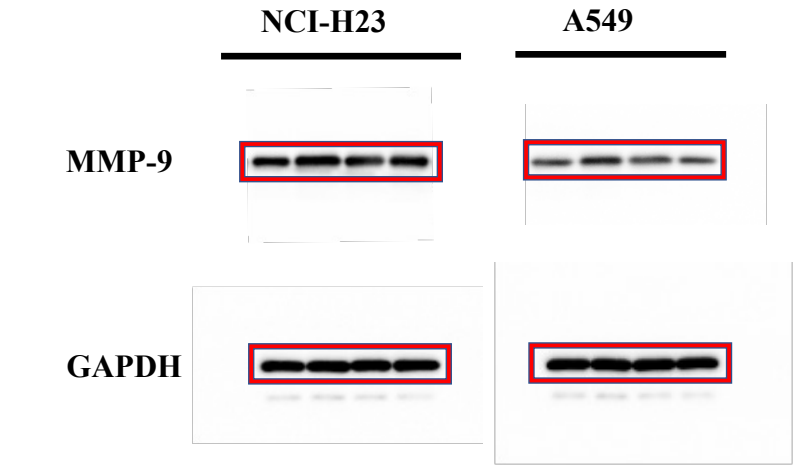

Supplement: Supplementary file 2 — Original Data File [file 41419_2023_6078_MOESM2_ESM.pdf]
